# Effects of the angiotensin-converting enzyme inhibitor captopril on occlusal-disharmony-induced cardiac dysfunction in mice

**DOI:** 10.1038/s41598-023-43099-6

**Published:** 2023-11-15

**Authors:** Aiko Ito, Yoshiki Ohnuki, Kenji Suita, Ichiro Matsuo, Misao Ishikawa, Takao Mitsubayashi, Yasumasa Mototani, Kenichi Kiyomoto, Michinori Tsunoda, Akinaka Morii, Megumi Nariyama, Yoshio Hayakawa, Hiroshi Tomonari, Satoshi Okumura

**Affiliations:** 1 Department of Orthodontics, Tsurumi University School of Dental Medicine, Yokohama, 230-8501 Japan; 2 Department of Physiology, Tsurumi University School of Dental Medicine, 2-1-3 Tsurumi, Tsurumi-ku, Yokohama, 230-8501 Japan; 3 Department of Periodontology, Tsurumi University School of Dental Medicine, Yokohama, 230-8501 Japan; 4 Department of Oral Anatomy, Tsurumi University School of Dental Medicine, Yokohama, 230-8501 Japan; 5 Department of Pediatric Dentistry, Tsurumi University School of Dental Medicine, Yokohama, 236-8501 Japan; 6 Department of Dental Anesthesiology, Tsurumi University School of Dental Medicine, Yokohama, 230-8501 Japan

**Keywords:** Physiology, Cardiology, Health care, Medical research

## Abstract

Occlusal disharmony is known to affect not only the oral cavity environment, but also the autonomic nervous system in the heart. Since the renin-angiotensin system (RAS) inhibitor captopril (Cap) is one of the first-line drugs for preventing cardiac remodeling in patients with heart failure, we hypothesized that Cap might prevent cardiac dysfunction induced by occlusal disharmony. Here, to test this idea, we used our bite-opening (BO) mouse model, which was developed by cementing a suitable appliance onto the mandibular incisor. Mice were divided into four groups: (1) Control, (2) BO, (3) Cap, and (4) BO + Cap. After 2 weeks, we evaluated cardiac function by echocardiography and confirmed that cardiac function was significantly decreased in the BO group compared to the control, while Cap ameliorated the dysfunction. Cardiac fibrosis, myocyte apoptosis and oxidative stress-induced myocardial damage in the BO group were significantly increased versus the control, and these increases were suppressed by Cap. Cardiac dysfunction induced by BO was associated with dual phosphorylation on PKCδ (Tyr-311/Thr-505), leading to activation of CaMKII with increased phosphorylation of RyR2 and phospholamban. Our results suggest that the RAS might play an important role in the development of cardiac diseases induced by occlusal anomalies.

## Introduction

The renin-angiotensin system (RAS) is a hormonal system that plays key roles not only in the regulation of arterial pressure and salt balance, but also in many physiological and pathophysiological mechanisms in almost every organ system^1^. The RAS system consists mainly of a two-step enzymatic cascade catalyzed by renin and angiotensin-converting enzyme (ACE), generating the bioactive peptide angiotensin II (Ang II). Ang II, the main RAS effector hormone, acts through two receptor types, Ang II type 1 and type 2 receptors (AT1R and AT2R), but most of the functions of Ang II are mediated by AT1R^2^.

AT1R actions include induction of reactive oxygen species (ROS) in cardiac myocytes^3^, induction of cardiac hypertrophy and myocyte apoptosis^4^, and stimulation of cardiac fibroblast proliferation and collagen synthesis^5^. Changes in the activity and responsiveness of AT1R occur with aging^6^ and might be associated with the development or progression of frailty^6^.

Oral frailty has been recently suggested as a novel construct defined as a decrease in oral function with a coexisting decline in cognitive and physical function, and is influenced by the number of natural teeth, chewing ability, articulatory oral motor skill, tongue pressure, and subjective difficulties in eating and swallowing^7,8^. Oral frailty might be an important contributor to loss of general health and physical weakening, such as physical frailty, sarcopenia, and subsequent disability^7^.

The Suita study, a cohort study of urban residents in Japan, demonstrated that occlusal disharmony, which was assessed using the Eichner index^9^, was significantly associated with increased risk of hypertension^10^, which is involved in the pathogenesis of cardiac dysfunction and its progression toward heart failure^11^.

Heart failure is a major cause of death throughout the world. Both the sympathetic nervous system (SNS)^12^ and the RAS system^6^ are activated in heart failure, and prolonged activation is inevitably detrimental^6,13–16^. Thus, worldwide standard guidelines for treating heart failure involve initiation of the chronically enhanced SNS activity, usually with β-blockers^12^ and RAS inhibitors^6^. There are many possibilities for interaction between the two systems, particularly when they are both in the activated, as in heart failure. Renin release from renal juxtaglomerular cells is stimulated by the SNS via β_1_-adrenoreceptors, which are involved in the pathophysiology of cardiovascular disease^17^.

**Figure 1:**
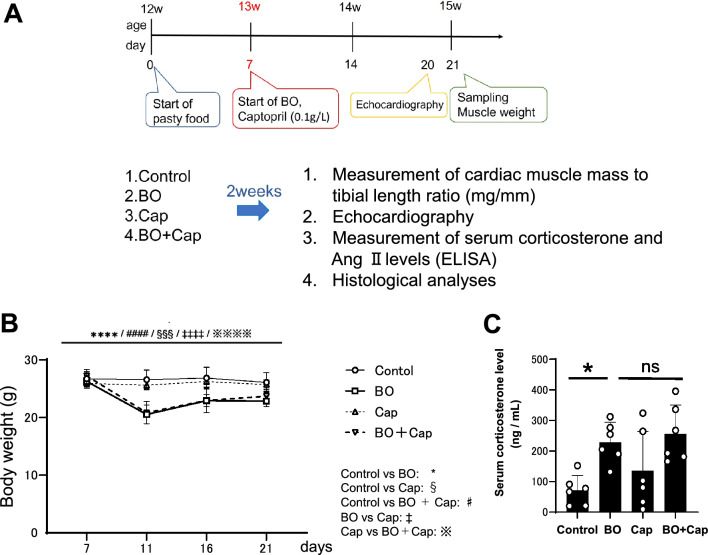
Experimental procedure and effects of BO on body weight and serum corticosterone levels. (**A**) Male 16-week-old C57BL/6 mice were divided into four groups: a normal control group (Control), a bite-opening (BO)-treated group, a captopril-treated group (Cap), and a BO plus Cap-treated (BO + Cap) group. (**B**) Body weight was measured daily for all animals throughout the 2-week experimental period. ^****^P < 0.0001 (Control vs. BO, ^####^*P* < 0.0001 (Control vs. BO + Cap, ^§§§^*P* < 0.001 (Control vs. Cap), ^‡‡‡‡^*P* < 0.0001 (BO vs. Cap), ^※※※※^*P* < 0.0001 (Cap vs. BO + Cap) by two-way repeated-measures ANOVA followed by the Bonferroni post hoc test (*n* = 5 each). (**C**) Serum corticosterone level was similarly and significantly increased after BO treatment in both the BO and BO + Cap groups (*n* = 6 each). *P < 0.05, **P < 0.01 by one-way ANOVA followed by the Tukey–Kramer post hoc test. Data are presented as mean ± SD and dots show individual data.

We recently examined the effects of occlusal disharmony on cardiac remodeling (fibrosis and myocyte apoptosis), cardiac function and susceptibility to atrial fibrillation in bite-opening (BO) mice, in which a 0.7-mm BO was introduced by cementing a suitable appliance onto the mandibular incisor^18–20^ (Fig. 1A). We found that BO treatment increased serum corticosterone, a key biomarker of stress, as well as the low-frequency/high-frequency ratio, an index of SNS activity^18^. In addition, BO-induced cardiac dysfunction and susceptibility to atrial fibrillation are ameliorated by propranolol, a non-selective β-adrenoreceptor antagonist (β-blocker)^18,19^.

We thus hypothesized that inhibition of RAS might prevent or reduce cardiac dysfunction induced by occlusal disharmony. Therefore, the aim of this study was to examine the effects of the ACE inhibitor captopril (Cap) on occlusal disharmony by evaluating cardiac function, histology, and signal transduction in the heart of Cap-treated BO mice, which have previously been used in research on occlusal disharmony^21–23^ (Fig. 1A).

## Results

### Effects of BO on body weight and serum corticostreole level with/without Cap treatment

Body weight (BW) in the control group showed no significant change during the experimental period (Fig. 1B). Conversely, BW gradually decreased in the BO and BO + Cap groups and reached a minimum at 4 days after the BO treatment, in accordance with previous findings by us^18,20^ and other groups^22,24^. After that, the BW of the BO and BO plus Cap groups (BO + Cap) gradually increased, but did not reach the preoperative level during the experimental period, in accordance with our previous findings^18,20^ (Fig. 1B).

Body weight loss is known to be closely associated with stress^25^. Comparison of the levels of serum corticosterone level, a key biomarker of stress, including that caused by BO treatment^18^, revealed a significant and similar increase of approximately 3.2-fold at 14 days after BO treatment in both the BO and BO + Cap groups (Fig. 1C).

These data suggest that the BO and BO + Cap groups are stressed similarly at 14 days after the BO treatment.

### Effects of BO on the consumption of food and drinking water with/without Cap treatment

We monitored the daily consumption of food and water, measured as an average of the animals in each cage, during the 2-week experimental period. Consumption level of food before and after BO treatment in the control and Cap groups were similar and did not show any significant change during the period (day 7 vs. day 21; Control: 10.5 ± 0.02 vs. 8.7 ± 0.3 g/day, *P* = NS, BO: 9.9 ± 1.1 vs. 9.9 ± 0.8 g/day,* P* = NS, Cap: 9.4 ± 0.9 vs. 8.5 ± 0.4 g/day, BO + Cap: 11.6 ± 3.5 vs. 10.9 ± 0.4 g/day,* P* < 0.01). (Supplementary Fig. 1A). Changes in the consumption of water showed a similar tendency (Supplementary Fig. 1B).

### Effects of BO on serum corticosterone levels with/without Cap treatment

Comparison of the levels of serum corticosterone level, a key biomarker of stress, including that caused by BO treatment^18^, revealed a significant and similar increase of approximately 3.2-fold at 14 days after BO treatment in both the BO and BO + Cap groups (Fig. 1C). These data suggest that the BO and BO + Cap groups are stressed similarly at 14 days after the BO treatment.

### Effects of BO on serum Ang II levels with/without Cap treatment

Sex, time-since-light-on, type of control, quantification technique (e.g. enzyme immunoassay or radioimmunoassay), sample matrix (e.g., plasma or urine) and quantification technique (e.g. enzyme assay or radioimmunoassay) can all significantly affected the basal corticosterone levels^26^, so that corticosterone is not necessarily a good biomarker of stress.

**Figure 2:**
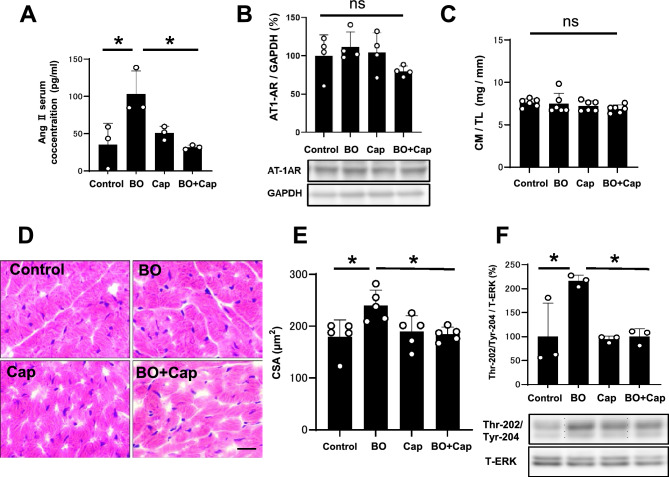
Effects of BO on the activation of RAS and cardiac hypertrophy. (**A**) Serum Ang II levels were significantly increased in the BO group compared to the control, and the increase was significantly suppressed by Cap (*n* = 3 each). *P < 0.05 by one-way ANOVA followed by the Tukey–Kramer post hoc test. (**B)** AT1 expression was similar in all four groups (*n* = 4 each). NS, not significantly different by one-way ANOVA followed by the Tukey–Kramer post hoc test. Images of full-size immunoblots are shown in Supplementary Fig. 2. (**C**) No significant difference in heart size in terms of cardiac muscle mass (CM) per tibial length ratio (TL) (mg/mm) at 2 weeks after BO treatment (*n* = 6 each). (**D** and** E**) Representative images of HE-stained sections of cardiac muscle in the Control (*upper left*), BO (*upper right*), Cap (*lower left*) and BO + Cap (*lower right*) groups. Scale bar = 20 μm (**D**). CSA, another index of hypertrophy, was slightly but significantly increased in the heart of BO mice, and the increase was suppressed by Cap (*n* = 5 each) (**E**). (**F**) Expression of ERK1/2 phosphorylation was significantly increased in the BO, and the increase was suppressed by Cap (*n* = 3 each). *P < 0.05 by one-way ANOVA followed by the Tukey–Kramer post hoc test. Images of full-size immunoblots are shown in Supplementary Fig. 3. Data are presented as mean ± SD and dots show individual data.

Activation of the RAS increases in response to chronic stress and Ang II might be classified as a stress hormone^27,28^. Thus, we examined serum Ang II levels by means of enzyme-linked immunosorbent assay (ELISA) at 14 days after the BO treatment. Serum Ang II levels were significantly increased in the BO group compared to the control (Control vs. BO: 35 ± 28 vs. 103 ± 31 pg/mL, *P* < 0.05 vs. Control), and the increase was significantly suppressed by Cap (BO vs. BO + Cap: 103 ± 31 vs. 32 ± 9 pg/mL, *P* < 0.05 vs. BO) (Fig. 2A).

These data support the idea that BO treatment significantly increased RAS activation and might be associated with the increase of serum Ang II levels in BO-treated mice. Cap alone had no effect on serum Ang II levels, but blocked the BO-induced increase.

### Effects of BO on AT1 expression with/without Cap treatment

Downregulation of the AT1 receptor was previously demonstrated to protect the heart from oxidative stress^29^. We thus examined the expression of AT1 receptors in the heart and found that they were similar among the four groups (Fig. 2B), suggesting that BO treatment did not alter AT1 receptor expression.

### Effects of BO on heart size and CSA with/without Cap treatment

We examined the effects of BO with/without Cap on heart size in terms of cardiac muscle mass per tibial length ratio (mg/g). Similar values were observed in all four groups (Fig. 2C).

We also examined myocyte cross-sectional area (CSA), another index of hypertrophy, and confirmed that CSA was slightly but significantly increased in the heart of BO mice (Control vs. BO; 180 ± 32 vs. 240 ± 30 μm^2^, *P* < 0.05 vs. Control), and the increase was suppressed by Cap (BO vs. BO + Cap; 240 ± 30 vs. 189 ± 31 μm^2^, *P* < 0.05 vs. BO) (Fig. 2D, E).

### Effects of BO on ERK1/2 phosphorylation with/without Cap treatment

In addition, we examined phosphorylation of extracellular signal-regulated kinase (ERK1/2) on Thr-202/Tyr-204 in the heart in the four groups because this might play an important role in the transduction of extracellular signals in hypertrophic responses induced by RAS activation, such as in cardiac myocytes^30^. ERK1/2 phosphorylation was significantly increased in the BO group (Control vs. BO; 100 ± 70 vs. 216 ± 12%, *P* < 0.05 vs. Control), and the increase was suppressed by Cap (BO vs. BO + Cap; 216 ± 12 vs. 100 ± 16%, *P* < 0.05 vs. BO) (Fig. 2F).

These data, together with previous findings^30^, suggest that BO treatment might slightly but significantly increase cardiac myocyte hypertrophy at least in part through RAS activation, even though the cardiac muscle mass per tibial length ratio was similar in the four groups.

### Effects of BO on cardiac function with/without Cap treatment

We also conducted echocardiography (Table 1) to evaluate cardiac function in terms of left ventricular ejection fraction (EF) and fractional shortening (%FS). Both parameters were significantly decreased in the BO group compared to the control (EF: Control vs. BO: 67 ± 1.6 vs. 60 ± 2.1%, *P* < 0.01; %FS: Control vs. BO: 32 ± 1.1 vs. 27 ± 1.3%, *P* < 0.01). Importantly, BO-mediated cardiac dysfunction was attenuated by pharmacological inhibition of RAS with Cap at 2 weeks (EF: BO vs. BO + Cap: 60 ± 2.1 vs. 66 ± 2.3%, *P* < 0.01; %FS: BO vs. BO + Cap: 27 ± 1.3 vs. 31 ± 1.6%, *P* < 0.01), with a decrease of left ventricular internal dimension at end-systole (BO vs. BO + Cap: 3.2 ± 0.2 vs. 3.0 ± 0.08 mm, *P* < 0.01).

**Table 1:** Cardiac function assessed by echocardiography with/without Cap.

	Control	BO	Cap	BO + Cap
n	6	10	6	10
EF	67 ± 1.6	60 ± 2.1**	66 ± 1.6	66 ± 2.3^##^
LVEDV	0.23 ± 0.02	0.21 ± 0.03	0.22 ± 0.02	0.20 ± 0.02*
LVESV	0.074 ± 0.009	0.086 ± 0.012	0.073 ± 0.006	0.066 ± 0.005^##^
%FS	32 ± 1.1	27 ± 1.3**	31 ± 1.1	31 ± 1.6^##^
LVIDd	4.5 ± 0.15	4.4 ± 0.18	4.4 ± 0.12	4.3 ± 0.14*
LVIDs	3.1 ± 0.1	3.2 ± 0.2	3.0 ± 0.08	3.0 ± 0.08^##^
HR	397 ± 61	373 ± 47	366 ± 52	364 ± 80
SV	0.15 ± 0.02	0.13 ± 0.02	0.14 ± 0.01	0.13 ± 0.02
CO	61 ± 11	48 ± 10	52 ± 11	48 ± 13
IVSTd	0.50 ± 0.04	0.48 ± 0.03	0.47 ± 0.06	0.47 ± 0.06
IVSTs	0.96 ± 0.05	0.84 ± 0.06	0.87 ± 0.05	0.86 ± 0.09
LVPWTd	0.50 ± 0.04	0.51 ± 0.06	0.52 ± 0.06	0.47 ± 0.05
LVPWTs	0.93 ± 0.04	0.82 ± 0.05**	0.88 ± 0.04	0.81 ± 0.04

EF (%), ejection fraction; LVEDV (mL), left ventricular end-diastolic volume; LVESV (mL), left ventricular end-systolic volume; FS (%), fractional shortening; LVDd (mm), left ventricular internal dimension at end-diastole; LVDs (mm), left ventricular internal dimension at end-systole; HR (bpm), heart rate; SV (mL), stroke volume; CO (mL/min), cardiac output; IVSTd (mm), interventricular septum thickness at end-diastole; IVSTs (mm), interventricular septum thickness at end-systole; LVPWTd (mm), posterior wall thickness at end-diastole; LVPWTs (mm), posterior wall thickness at end-systole.

*P < 0.05, **P < 0.01 vs. Control.

^#^*P* < 0.05, ^##^*P* < 0.01 vs. BO.

In addition, left ventricular end-systolic volume (LVESV) in the BO group tended to be increased (not significant) compared to the control, and LVESV and left ventricular internal dimension at end-systole (LVDs) were significantly reduced in the BO + Cap group, compared to the BO group.

These data suggest that BO treatment might mediate systolic dysfunction through the activation of RAS.

### Effects of BO on cardiac function with/without losartan treatment

We also examined cardiac function with/without angiotensin receptor blocker (ARB) losartan to evaluate cardiac function in terms of EF and %FS by echocardiography (Table 2). Both parameters were significantly decreased in the BO group compared to the control (LVEF: Control vs. BO: 67 ± 2.0 vs. 60 ± 4.4%, *P* < 0.01; %FS: Control vs. BO: 32 ± 1.4 vs. 28 ± 2.8%, *P* < 0.01). Again, BO-mediated cardiac dysfunction was attenuated by pharmacological inhibition of RAS with losartan at 2 weeks (EF: BO vs. BO + Cap: 60 ± 4.4 vs. 67 ± 1.3%, *P* < 0.01; %FS: BO vs. BO + Cap: 28 ± 2.8 vs. 32 ± 0.9%, *P* < 0.01).

**Table 2:** Cardiac function assessed by echocardiography with/without losartan**.**

	Control	BO	losartan	BO + losartan
n	6	7	6	5
EF	67 ± 2.0	60 ± 4.4**	66 ± 1.4	67 ± 1.3^##^
LVEDV	0.23 ± 0.01	0.21 ± 0.02	0.22 ± 0.01	0.20 ± 0.01
LVESV	0.074 ± 0.01	0.085 ± 0.02	0.077 ± 0.006	0.066 ± 0.003^#^
%FS	32 ± 1.4	28 ± 2.8**	31 ± 0.9	32 ± 0.9^##^
LVIDd	4.5 ± 0.09	4.4 ± 0.16	4.5 ± 0.07	4.3 ± 0.07
LVIDs	3.1 ± 0.1	3.2 ± 0.2	3.1 ± 0.09	3.0 ± 0.04^##^
HR	428 ± 37	402 ± 28	434 ± 16	412 ± 62
SV	0.15 ± 0.01	0.13 ± 0.01**	0.15 ± 0.01	0.13 ± 0.01
CO	59 ± 5.6	46 ± 4.8**	57 ± 2.1	51 ± 5.0
IVSTd	0.49 ± 0.04	0.50 ± 0.03	0.52 ± 0.04	0.50 ± 0.05
IVSTs	0.88 ± 0.05	0.83 ± 0.03	0.89 ± 0.04	0.90 ± 0.05
LVPWTd	0.50 ± 0.03	0.49 ± 0.04	0.50 ± 0.04	0.52 ± 0.02
LVPWTs	0.98 ± 0.02	0.83 ± 0.06**	0.95 ± 0.04	0.91 ± 0.05^#^

EF (%), ejection fraction; LVEDV (mL), left ventricular end-diastolic volume; LVESV (mL), left ventricular end-systolic volume; FS (%), fractional shortening; LVIDd (mm), left ventricular internal dimension at end-diastole; LVIDs (mm), left ventricular internal dimension at end-systole; HR (bpm), heart rate; SV (mL), stroke volume; CO (mL/min), cardiac output; IVSTs (mm), interventricular septum thickness at end-systole; IVSTd (mm), interventricular septum thickness at end-diastole; LVPWTd (mm), posterior wall thickness at end-diastole; LVPWTs (mm), posterior wall thickness at end-systole.

*P < 0.05, **P < 0.01 vs. Control.

^#^*P* < 0.05, ^##^*P* < 0.01 vs. BO.

These data, together with the data with/without Cap, suggest that BO treatment might mediate cardiac dysfunction through the activation of RAS.

### Effects of BO on cardiac fibrosis with/without Cap treatment

**Figure 3:**
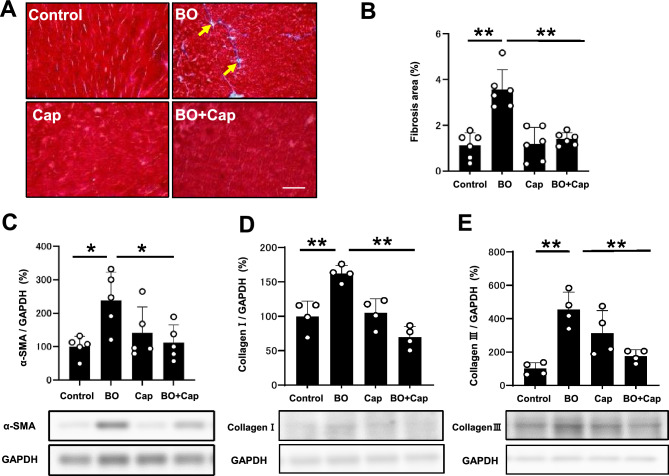
Effects of Cap on BO-induced fibrosis in the heart. (**A**) Representative images of Masson-trichrome-stained sections of cardiac muscle in the Control (upper left), BO (upper right), Cap (lower left) and BO + Cap (lower right) groups. Scale bar = 100 μm. (**B**) The area of fibrosis was significantly increased in the BO group (*n* = 6), but this increase was blocked in the BO + Cap group (*n* = 6 each). **P < 0.01 by one-way repeated-measures ANOVA followed by the Tukey–Kramer post hoc test. (**C**) Expression of α-SMA, a fibrosis-related gene, was significantly increased in the BO group (*n* = 5), but this increase was blocked in the BO + Cap group (*n* = 5). *P < 0.05 by one-way ANOVA followed by the Tukey–Kramer post hoc test. Data are presented as mean ± SD and dots show individual data. Images of full-size immunoblots are presented in Supplementary Fig. 4. (**D** and **E**) Expressions of collagen 1 (**D**) and collagen 3 (**E**) was significantly increased in the BO group, but these increases were blocked in the BO + Cap group (*n* = 4 each). *P < 0.05 by one-way ANOVA followed by the Tukey–Kramer post hoc test. Data are presented as mean ± SD and dots show individual data. Images of full-size immunoblots are presented in Supplementary Figs. 5, 6.

We examined the effects of BO treatment with/without Cap on fibrosis in cardiac muscle by means of Masson-trichrome staining (Fig. 3A). BO treatment significantly increased the area of fibrosis in cardiac muscle (Control vs. BO: 1.1 ± 0.6 vs. 3.6 ± 0.9%, *P* < 0.01 by one-way ANOVA followed by the Tukey–Kramer post hoc test), in accordance with our previous findings^18,20^ (Fig. 3B). Cap alone did not alter the area of fibrosis, but it blocked the BO-induced increase of fibrosis (BO vs. BO + Cap: 3.6 ± 0.8 vs. 1.4 ± 0.3%, *P* < 0.01 by one-way ANOVA followed by the Tukey–Kramer post hoc test) (Fig. 3B).

### Effects of BO on α-SMA expression with/without Cap treatment

We also evaluated cardiac fibrosis by measuring the level of α-smooth muscle actin (α-SMA) expression at 2 weeks after the start of BO, because this parameter is known to be associated with cardiac fibrosis^20^. Expression of α-SMA was significantly increased in cardiac muscle of BO mice (Control vs. BO: 100 ± 31 vs. 238 ± 85%, *P* < 0.05 by one-way ANOVA followed by the Tukey–Kramer post hoc test), and the increase was significantly suppressed by Cap (BO vs. BO + Cap: 238 ± 85 vs. 112 ± 52%, *P* < 0.05 by one-way ANOVA followed by the Tukey–Kramer post hoc test) (Fig. 3C).

These data support the idea that cardiac fibrosis induced by BO might be mediated, at least in part through the activation of RAS.

### Effects of BO on collagen 1 and 3 protein expression with/without Cap treatment

We examined the protein expression of collagen I (Fig. 3D) and collagen III (Fig. 3E) in the heart in the four groups. Their expression levels were significantly increased in the heart of BO-treated mice (collagen 1: Control vs. BO: 100 ± 22 vs. 162 ± 12%, *P* < 0.01 by one way ANOVA followed by the Tukey–Kramer post hoc test; collagen III: Control vs. BO: 100 ± 36 vs. 455 ± 103%, *P* < 0.01 by one way ANOVA followed by the Tukey–Kramer post hoc test) (Fig. 3D, E). Cap alone did not alter the expression of collagen I and collagen III, but blocked the BO-induced increases of collagen I and collagen III (collagen 1: BO vs. BO + Cap: 162 ± 12% vs. 70 ± 16%, *P* < 0.01 by one way ANOVA followed by the *Tukey–Kramer *post hoc test; collagen 3: BO vs. BO + Cap: 455 ± 103 vs. 175 ± 36, *P* < 0.01 by one way ANOVA) (Fig. 3D, E).

These data suggest that protein expression of collagen I and collagen III induced by BO might be mediated, at least in part through the activation of the RAS.

### Effects of BO on cardiac apoptosis with/without Cap treatment

**Figure 4:**
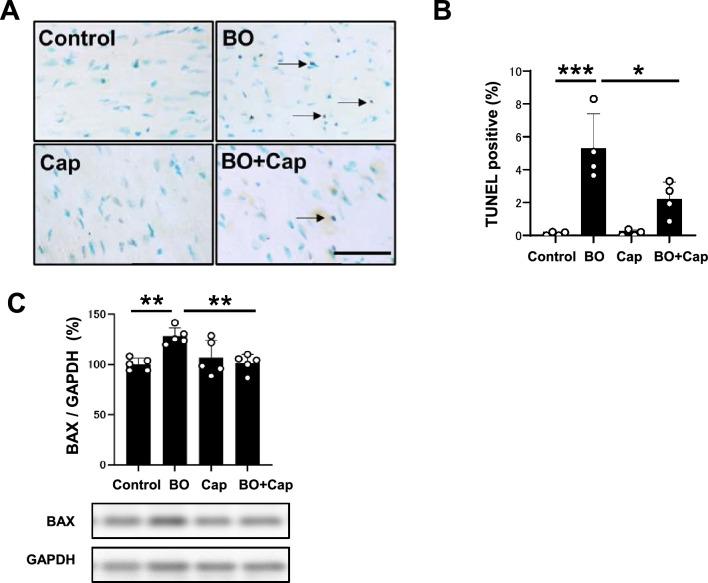
Effects of Cap on BO-induced cardiac myocyte apoptosis. (**A**) TUNEL-positive nuclei (black arrows) in representative TUNEL-stained sections were counted in cardiac muscle in the Control (upper left), BO (upper right), captopril (Cap; lower left) and BO + Cap (lower right) groups. Scale bar = 5 μm. (**B**) The number of TUNEL-positive nuclei was significantly increased in the BO group (*n* = 4), but this increase was blocked in the BO + Cap group (*n* = 4). Data are presented as mean ± SD and dots show individual data. *P < 0.05, ***P < 0.001 by one-way ANOVA followed by the Tukey–Kramer post hoc test. (**C**) Bax expression was significantly increased in the BO group (*n* = 5), but this increase was blocked in the BO + Cap group (*n* = 5 each). **P < 0.01 by one-way ANOVA followed by the Tukey–Kramer post hoc test. Data are presented as mean ± SD and dots show individual data. Images of full-size immunoblots are presented in Supplementary Fig. 7.

We next evaluated cardiac myocyte apoptosis in BO mice with/without Cap treatment by means of terminal deoxyribonucleotidyl transferase (TdT)-mediated biotin-16-deoxyuridine triphosphate (dUTP) nick-end labeling (TUNEL) (Fig. 4A). BO treatment significantly increased cardiac myocyte apoptosis (Control vs. BO: 0.1 ± 0.12 vs. 5.3 ± 2.1%, *P* < 0.001 vs. Control). Cap alone had no effect on the number of TUNEL-positive cardiac myocytes, but it blocked the BO-induced increase of TUNEL-positive cardiac myocytes (BO vs. BO + Cap; 5.3 ± 2.1% vs. 2.20 ± 1.1%, *P* < 0.05 vs. BO) (Fig. 4B).

BO treatment significantly increased expression of B cell lymphoma 2 associated X (Bax), an accelerator of apoptosis, in the heart of BO mice (Control vs. BO; 100 ± 6.1 vs. 128 ± 8.4%, *P* < 0.01 vs. Control) in accordance with a previous study^18^. Cap alone had no effect on Bax expression, but blocked the BO-induced increase (BO vs. BO + Cap; 128 ± 8.4 vs. 101 ± 8.3%, *P* < 0.01 vs. BO) (Fig. 4C).

### Effects of BO on oxidative stress with/without Cap treatment

**Figure 5:**
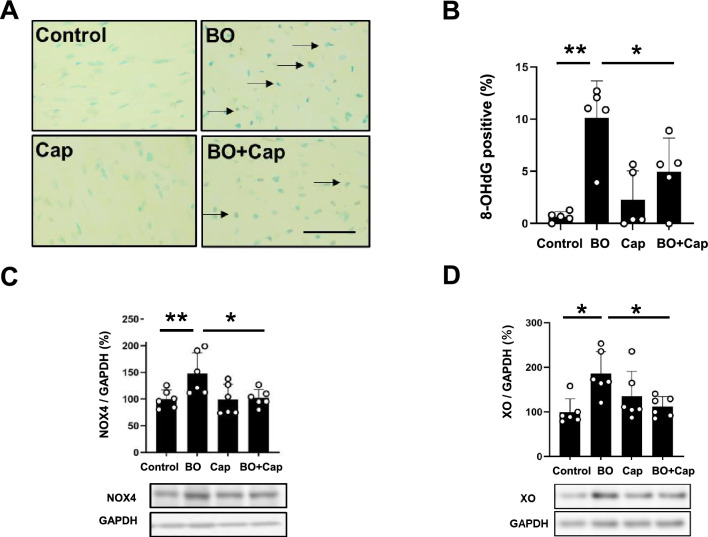
Effects of Cap on BO-induced oxidative stress in cardiac muscle. (**A**) Representative immunohistochemical images of oxidative DNA damage (8-OHdG) in cardiac muscle in the Control (upper left), BO (upper right), captopril (Cap; lower left) and BO + Cap (lower right) groups. Scale bar = 5 μm. (**B**) 8-OHdG-positive nuclei were significantly increased in the BO group (*n* = 5), but this increase was blocked in the BO + Cap group (*n* = 5). *P < 0.05, **P < 0.01 by one-way ANOVA followed by the Tukey–Kramer post hoc test. (**C**) NOX4 expression was significantly increased in the BO group (*n* = 6), and this increase was significantly blocked in the BO + Cap group (*n* = 6). *P < 0.05 by one-way ANOVA followed by the Tukey–Kramer post hoc test. Data are presented as mean ± SD and dots show individual data. Images of full-size immunoblots are shown in Supplementary Fig. 9. (**D**) Expression of XO was significantly increased in the BO group (*n* = 6), and this increase was significantly blocked in the BO + Cap group (*n* = 6). *P < 0.05 by one-way ANOVA followed by the Tukey–Kramer post hoc test. Data are presented as mean ± SD and dots show individual data. Images of full-size immunoblots are shown in Supplementary Fig. 10.

We evaluated oxidative stress in the myocardium by means of 8-hydroxy-2’-deoxyguanosine (8-OHdG) immunostaining (Fig. 5A).

We first examined the validity of the 8-OHdG immunostaining used in this experiment by incubation in TBS-T with (positive control) or without (negative control) 0.3% H_2_O_2_ at room temperature in TBS-T for 1 h at room temperature before anti-8-OHdG antibody treatment and confirmed that the 8-OHdG antibody staining procedure could distinguished 8-OHdG-positive from non-positive nuclei (Supplementary Fig. 8).

The ratio of 8-OHdG-positive/total cardiac myocytes was significantly increased in the BO mice (Control vs. BO; 0.7 ± 0.5 vs. 10.1 ± 3.5%, *P* < 0.01 vs. Control), and the increase was suppressed by Cap (BO vs. BO + Cap; 0.7 ± 0.5 vs. 5.0 ± 3.2%, *P* < 0.05 vs. BO) (Fig. 5B).

These data, together with the data shown in Figs. 3, 4 and Table 1, suggest that oxidative stress-induced myocardial damage might be increased at least in part through the activation of RAS, which might contribute to the cardiac remodeling and dysfunction in BO mice.

### Effects of BO on NOX4 and XO expression with/without Cap treatment

AT1 stimulation produces cardiac ROS generation through a number of pathways, including nicotinamide adenine dinucleotide phosphate oxidase 4 (NOX4) and xanthine oxidase (XO), and may be involved in myocardial fibrosis and cardiac remodeling^31^.

Two NOX isoforms, NOX2 and NOX4, are expressed in the heart, and their activity is regulated by their expression level^32^. Importantly, AT1-induced cardiac fibrosis and remodeling might be caused by ROS production via AT1/NOX4 interaction^31^. We therefore compared NOX4 protein expression in the heart among the four groups. NOX4 expression was significantly increased in the BO-group (Control vs. BO; 100 ± 17 vs. 148 ± 39%, *P* < 0.01 vs. Control), and the increase was suppressed by Cap (BO vs. BO + Cap; 148 ± 39 vs. 102 ± 16%, *P* < 0.05 vs. BO) (Fig. 5C).

We next examined XO expression in the heart in the four groups. XO expression was significantly increased in the BO-group (Control vs. BO; 100 ± 29 vs. 186 ± 49%, *P* < 0.05 vs. Control), and the increase was suppressed by Cap (BO vs. BO + Cap; 186 ± 49 vs. 112 ± 22%, *P* < 0.05 vs. BO) (Fig. 5D).

These data suggest that BO-induced ROS production might contribute at least in part to the upregulation of NOX4 and XO.

### Effects of BO on PKCδ phosphorylation with/without Cap treatment

Protein kinase C (PKC) δ, but not other PKC isoforms, is important for the development of AT1-induced cardiac remodeling^33^. In addition, dual phosphorylation of PKCδ on Tyr-311/Thr-505 is known to occur and accumulate during oxidative stress^34^.

**Figure 6:**
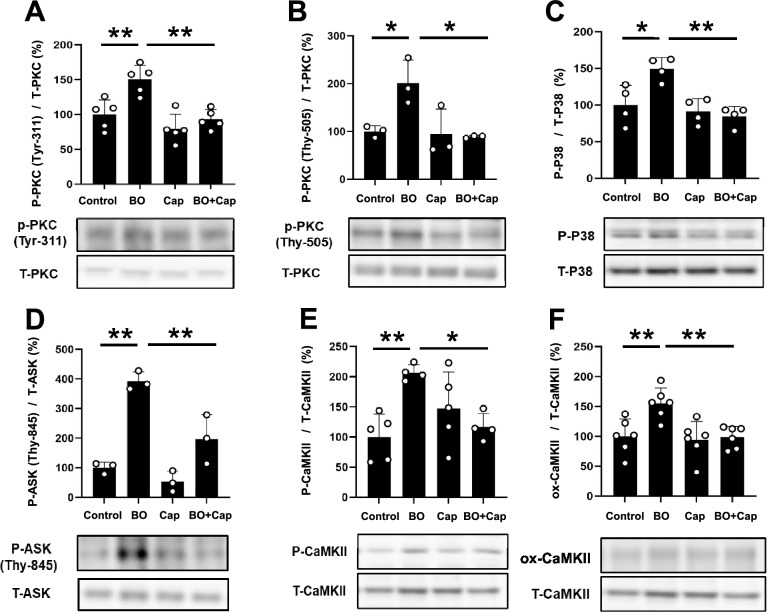
Effects of Cap on BO-induced phospho-PKC, phospho-p38, phospho-ASK, phospho-CaMKII and ox-CaMKII in the heart of BO mice. (**A**) Expression of phospho-PKC (Tyr-311) was significantly increased in the BO group (*n* = 5), but this increase was significantly blocked in the BO + Cap group (*n* = 5). **P < 0.01 by one-way ANOVA followed by the Tukey–Kramer post hoc test. Images of full-size immunoblots are shown in Supplementary Fig. 11. (**B**) Expression of phospho-PKC (Thy-505) was significantly increased in the BO group (*n* = 3), but this increase was significantly blocked in the BO + Cap group (*n* = 3). *P < 0.05 by one-way ANOVA followed by the Tukey–Kramer post hoc test. Images of full-size immunoblots are shown in Supplementary Fig. 12. (**C**) Expression of phospho-p38 (Thr180/Tyr182) was significantly increased in the BO group (*n* = 4), but this increase was blocked in the BO + Cap group (*n* = 4). *P < 0.05, **P < 0.01 by one-way ANOVA followed by the Tukey–Kramer post hoc test. Images of full-aize immunoblots are presented in Supplementary Fig. 13. (**D**) Expression of phospho-ASK (Thy-845) was significantly increased in the BO group (*n* = 3), but this increase was significantly blocked in the BO + Cap group (*n* = 3). **P < 0.01 by one-way ANOVA followed by the Tukey–Kramer post hoc test. Images of full-size immunoblots are shown in Supplementary Fig. 14. (**E**) Expression of phospho-CaMKII (Thr-286) was significantly increased in the BO group (*n* = 5), but this increase was significantly blocked in the BO + Cap group (*n* = 5). *P < 0.05, **P < 0.01 by one-way ANOVA followed by the Tukey–Kramer post hoc test. Images of full-size immunoblots are shown in Supplementary Fig. 15. (**F**) Expression of ox-CaMKII was significantly increased in the BO group (*n* = 6), but this increase was significantly blocked in the BO + Cap group (*n* = 6). **P < 0.01 by one-way ANOVA followed by the Tukey–Kramer post hoc test. Images of full-size immunoblots are shown in Supplementary Fig. 16. Data are presented as mean ± SD and dots show individual data.

We therefore examined the phosphorylation status of PKCδ on tyrosine 311 and threonine 505 in the heart in the four groups. Phosphorylation of PKCδ on tyrosine 311 was significantly increased in the BO-group (Control vs. BO; 100 ± 21 vs. 150 ± 20%, *P* < 0.01 vs. Control), and the increase was suppressed by Cap (BO vs. BO + Cap; 150 ± 20 vs. 93 ± 14%, *P* < 0.01 vs. BO) (Fig. 6A). In addition, phosphorylation of PKCδ on threonine 505 was significantly increased in the BO-group (Control vs. BO; 100 ± 12 vs. 202 ± 48%, *P* < 0.05 vs. Control), and the increase was suppressed by Cap (BO vs. BO + Cap; 202 ± 48 vs. 89 ± 2.7%, *P* < 0.05 vs. BO) (Fig. 6B).

These data, together with the data shown in Fig. 5, suggest that BO-induced oxidative stress might be mediated by AT1-mediated dual phosphorylation of PKCδ on tyrosine 311 and threonine 505, leading to ROS production through the upregulation of NOX4 and XO.

### Effects of BO on p38 phosphorylation with/without Cap treatment

Overexpression of constitutively active PKCδ induces phosphorylation of the p38 mitogen-activated protein kinase (MAPK) signaling pathway and apoptosis of cardiac myocytes^35^. In addition, ROS derived from NOX4 and XO induces oxidative stress and subsequently activates p38, leading to cardiac remodeling and dysfunction^20^.

We therefore examined the phosphorylation levels of p38 in the heart in the four groups. Phosphorylation of p38 on Thr-180/Tyr-182 was significantly increased in the BO-group (Control vs. BO; 100 ± 27 vs. 149 ± 16%, *P* < 0.05 vs. Control), and the increase was suppressed by Cap (BO vs. BO + Cap; 149 ± 16 vs. 85 ± 14%, *P* < 0.01 vs. BO) (Fig. 6C).

These data suggest that excessive ROS production derived from BO-induced upregulation of NOX4 and XO activates the p38 MAPK signaling pathway at least in part through the activation of RAS, which might cause cardiac remodeling and dysfunction in accordance with our previous study^20^.

### Effects of BO on ASK phosphorylation with/without Cap treatment

ROS production was recently demonstrated to increase activation of ASK1, which signals selectively to p38 MAPK and orchestrates cardiac remodeling in response to stress, including BO treatment^20^. We therefore examined the phosphorylation levels of ASK1 in the heart in the four groups. Phosphorylation of ASK1 on threonine 845 was significantly increased in the BO-group (Control vs. BO; 100 ± 19 vs. 392 ± 32%, *P* < 0.01 vs. Control), and the increase was suppressed by Cap (BO vs. BO + Cap; 392 ± 32 vs. 197 ± 83%, *P* < 0.01 vs. BO) (Fig. 6D).

These data suggest that BO treatment might activate the ASK1-p38 MAPK cascade at least in part through the activation of RAS, leading to cardiac remodeling and dysfunction.

### Effects of BO on CaMKII phosphorylation and oxidation with/without Cap treatment

Calmodulin kinase II (CaMKII) is activated via phosphorylation and oxidation in the presence of ROS and contributes to the development of cardiac remodeling and dysfunction^36^. We thus examined the amounts of phospho-CaMKII (Thr-286) (Fig. 6E) and oxidized methionine-281/282 CaMKII (ox-CaMKII) (Fig. 6F) in the heart in the four groups and found that they were significantly increased in the BO (phospho-CaMKII [Thr-286]: Control vs. BO; 100 ± 38 vs. 195 ± 28%, *P* < 0.01 vs. Control; ox-CaMKII: Control vs. BO; 100 ± 29 vs. 155 ± 26%, *P* < 0.01 vs. Control). The increase was suppressed by Cap (phospho-CaMKII (Thr-286): BO vs. BO + Cap; 195 ± 28 vs. 119 ± 20%, *P* < 0.05 vs. BO; ox-CaMKII: BO vs. BO + Cap; 155 ± 26% vs. 99 ± 18%, *P* < 0.01 vs. BO) **(**Fig. 6E and F). Note that the size of ox-CaMKII (approximately 60 kDa) is different from that of the major band of t-CaMKII (approximately 50 kDa) in accordance with previous findings by us^19^ and another group (Supplementary Fig. 16)^37^.

These data suggest that CaMKII signaling might be activated by BO treatment through NOX4- and XO-mediated generation of ROS at least in part via the activation of RAS.

### Effects of BO on PLB and RyR2 phosphorylation with/without Cap treatment

Since phosphorylation of most Ca^2+^-handling proteins has been shown to be altered in many models of experimental heart failure, which might lead to increased Ca^2+^ leakage, we next examined the effects of BO treatment on phospholamban (PLB) phosphorylation at Thr-17 and Ser-16, and RyR2 phosphorylation at Ser-2814 and Ser-2808. These phosphorylations are mediated by CaMKII and protein kinase A, respectively ^16^.

**Figure 7:**
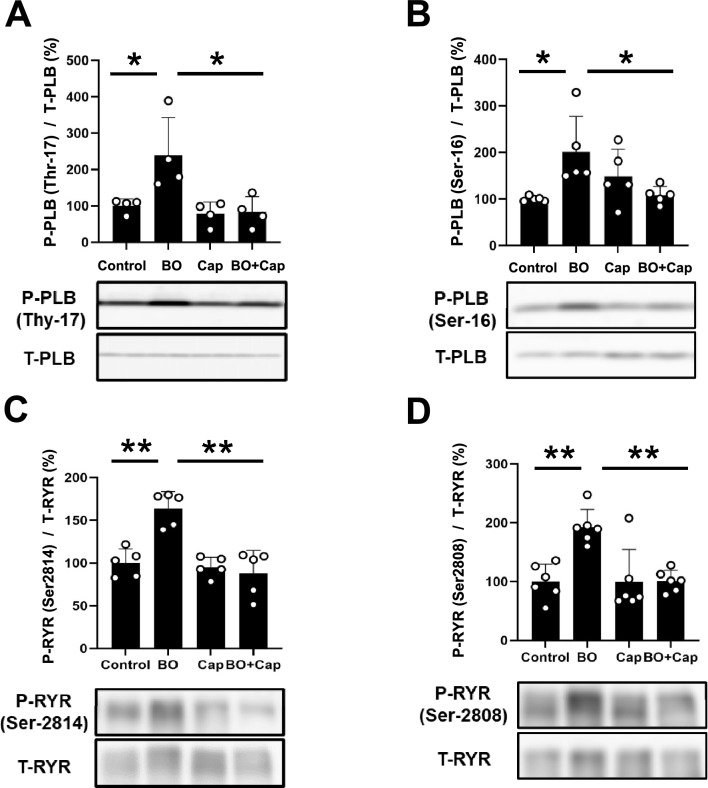
Effects of Cap on BO-induced phospho-PLB and phospho-RyR2 in the heart of BO mice. (**A**) Expression of phospho-PLB (Thr-17) was significantly increased in the BO group (*n* = 4), but this increase was blocked in the BO + Cap group (*n* = 4). *P < 0.05 by one-way ANOVA followed by the Tukey–Kramer post hoc test. Images of full-size immunoblots are shown in Supplementary Fig. 17. (**B**) Expression of phospho-PLB (Ser-16) was significantly increased in the BO group (*n* = 5), but this increase was significantly blocked in the BO + Cap group (*n* = 5). *P < 0.05 by one-way ANOVA followed by the Tukey–Kramer post hoc test. Images of full-size immunoblots are shown in Supplementary Fig. 18. (**C**) Expression of phospho-RyR2 (Ser-2814) was significantly increased in the BO group (*n* = 5), but this increase was blocked in the BO + Cap group (*n* = 5). **P < 0.01 by one-way ANOVA followed by the Tukey–Kramer post hoc test. Data are presented as mean ± SD and dots show individual data. Images of full-size immunoblots are presented in Supplementary Fig. 19. (**D**) Expression of phospho-RyR2 (Ser-2808) was significantly increased in the BO group (*n* = 6), but this increase was blocked in the BO + Cap group (*n* = 6). **P < 0.01 by one-way ANOVA followed by the Tukey–Kramer post hoc test. Data are presented as mean ± SD and dots show individual data. Images of full-size immunoblots are shown in Supplementary Fig. 20.

Phospho-PLB (Thr-17) was significantly increased in the heart of BO-mice (Control vs. BO; 100 ± 19 vs. 239 ± 104%, *P* < 0.05 vs. Control) (Fig. 7A). This increase was significantly attenuated by Cap (BO vs. BO + Cap; 239 ± 104 vs. 83 ± 42%, *P* < 0.05 vs. BO) (Fig. 7A).

Phospho-PLB (Ser-16) was also significantly increased in the heart of BO mice (Control vs. BO; 100 ± 5.5 vs. 148 ± 59%, *P* < 0.05 vs. Control). Again, the increase was significantly attenuated by Cap (BO vs. BO + Cap; 148 ± 59 vs. 108 ± 19%, *P* < 0.05 vs. BO) (Fig. 7B).

Phospho-ryanodine receptor 2 (RyR2) (Ser-2814) was significantly increased in the heart of BO mice (Control vs. BO; 100 ± 16 vs. 164 ± 20%, *P* < 0.01 vs. Control) (Fig. 7C), and this increase was significantly attenuated by Cap (BO vs. BO + Cap; 164 ± 20 vs. 88 ± 27%, *P* < 0.01 vs. BO) (Fig. 7C).

Phospho-RyR2 (Ser-2808) was also significantly increased in the heart of BO mice (Control vs. BO; 100 ± 30 vs. 193 ± 30%, *P* < 0.01 vs. Control) (Fig. 7D). Again, this increase was significantly attenuated by Cap (BO vs. BO + Cap; 193 ± 30 vs. 101 ± 18%, *P* < 0.01 vs. BO) (Fig. 7D).

These data, together with previous findings^38,39^, suggest that BO might increase PLB phosphorylatin at least in part through the activation of RAS, leading to ROS-mediated elevation of diastolic sarcoplasmic reticulum Ca^2+^ leakage in cardiac myocytes.

## Discussion

Poor oral status, as determined by the number of natural teeth, chewing ability, articulatory oral motor skill, tongue pressure, and subjective difficulties in eating and swallowing, might predict future physical weakening, such as onsets of physical frailty and disability^8^. Patients with poor oral health have higher incidences of comorbidities, such as cardiovascular disease and stroke^40^, chronic obstructive pulmonary disease^41^, and diabetes mellitus^42^. More importantly, poor oral health is associated with mortality when accompanied by occlusal disharmony due to tooth loss and periodontal disease (a principal component of poor oral health)^43,44^. Therefore, prevention of poor oral health at an earlier stage might be important for improving or maintaining physical abilities, including the status of the cardiovascular system.

Occlusal disharmony is induced by either loss or incorrect positioning of teeth^45^, and leads to morphological changes such as cardiac fibrosis, myocyte apoptosis and myocyte oxidative DNA damage, eventually resulting in cardiac dysfunction and susceptibility to atrial fibrillation, as previously demonstrated in BO mice by our group^18,19^.

We demonstrated in 2020 that 2-week occlusal disharmony causes cardiac dysfunction in bite-opening mice^18^, which have previously been used in research on occlusal disharmony^21,22,46^. However, the mechanism is not yet clearly understood.

In the previous study^18^, we carried out HRV analysis and compared the low frequency (LF)/high frequency (HF) ratio, an index of the sympathetic nervous activity^47^, normalized HF (nHF), an index of the parasympathetic nervous activity, and standard deviation of normal R-R intervals (SDNN), which is a measure of total autonomic instability^47–49^. The LF/HF ratio was significantly increased and nHF was significantly decreased, compared to the baseline at all time points after BO, as expected, because occlusal disharmony increases stress in humans^50^ and in rats^51^. However, mean HR was not increased in BO mice compared to the control, as in the case of the present study study.

Recently, it has been demonstrated that rats exposed to stress exhibit significantly increased serum corticosterone levels (> 200 ng/mL from baseline) to the same degree as BO mice, but do not show increased HR, compared to the baseline, even if the LF/HF ratio is increased and nHF is decreased, as observed in BO mice^52^. SDNN reflects the balance between the sympathetic and parasympathetic inputs to the cardiac pacemaker and thus SDNN is also a measure of total autonomic instability^47–49^. We do not completely understand the mechanisms that contribute to the lack of increase of HR after BO treatment. However, since behavioral and physiological flexibility to respond to stress depend upon parasympathetic modulation, autonomic imbalance might be augmented in BO mice, leading to the lack of increase of HR after BO treatment^18,47,48^.

In this study, we found that RAS system was activated in BO mice, as in the case of pressure-induced cardiac dysfunction induced by transverse aortic constriction (TAC) in mice^53^, while cardiac function declined at 2 weeks after BO treatment. It has been reported that cardiac dysfunction might be induced at 1 week after chronic sympathetic activation with isoproterenol, but might take 3 weeks to appear after TAC surgery in mice^14–16^.

Considering that cardiac function declines at 2 weeks after BO treatment, our current study, together with the previous studies, might suggest that activation of the SNS and the RAS contribute in concert to the development of cardiac dysfunction induced by occlusal disharmony.

PKC consists of a family of at least 11 isoforms and PKC isozymes are expressed differentially in various organs and tissues^54^. The distribution of PKC isoforms in the heart has been studied in several species and Ca^2+^-independent PKC isoforms PKCδ and PKCε are predominantly expressed in murine heart^55^. Ang II mediates various PKC-signaling-related cellular functions associated with cardiovascular function and disease^56^. However, the roles of PKCε and PKCδ are temporally distinct and opposing: PKCε plays a key role in cardioprotection as a result of intracellular translocation from cytosol to the membrane fraction^57^. On the other hand, PKCδ remains in the cytosol and, upon phosphorylation at Tyr-311 and Thr-505, plays a role in promoting stress-mediated cardiac fibrosis, myocyte apoptosis and myocyte oxidative DNA damage^33,58^.

It is well known that enhanced RAS function results in a marked activation of PKC^59^, resulting in increased ROS production and abnormal Ca^2+^ release from the SR. These changes are associated with hyperphosphorylation of RyR2 (Ser-2808) and PLB (Ser-16), which were originally identified as protein kinase A-dependent phosphorylation sites^60^. Therefore, we examined the phosphorylation levels of PKCδ at Tyr-311 and Thr-505 and confirmed that phosphorylation at both sites was significantly increased in the heart of BO mice. Furthermore, this increase was significantly attenuated by Cap. These data suggest that modulation of Ang II via AT1R-mediated PKCδ phosphorylation might be important for the development of occlusal disharmony-induced cardiac remodeling and dysfunction in BO mice.

**Figure 8:**
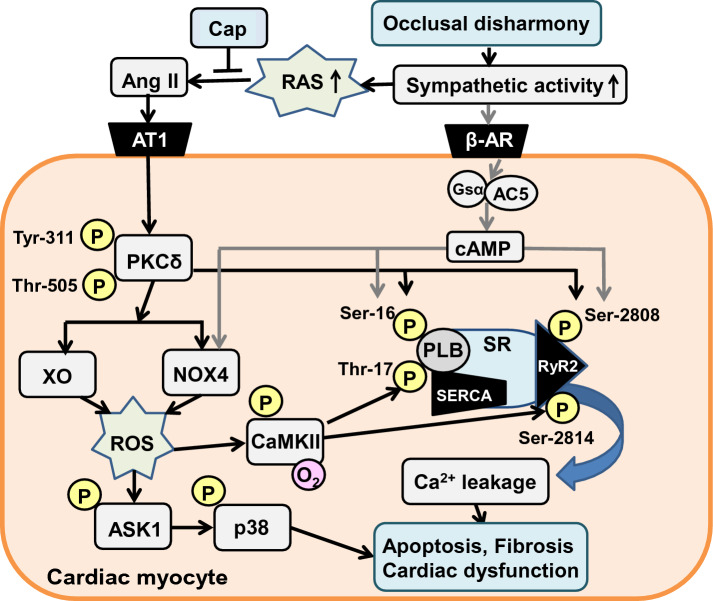
This scheme illustrates the proposed role of RAS in the heart of BO-treated mice. BO-treatment induces dual phosphorylation on PKCδ (Tyr-311/Thr-505), leading to CaMKII activation with the increased ROS production via activation of NOX4/XO and the ASK1-p38 MAPK cascade, which mediates the phosphorylation of RyR2 (Ser-2814) and PLB (Thr-17). BO-treatment was previously reported to induce sympathetic nerve activity by our group, leading to the phosphorylation of RyR2 (Ser-2808) and PLB (Ser-16)^18^. These changes might cause Ca^2+^ leakage, leading to fibrosis, myocyte apoptosis, oxidative stress and cardiac dysfunction. Solid black lines represent findings in this study and solid gray lines represents finding reported previously^18,20^. β-AR, β-adrenoreceptor; Gsα, α subunit of Gs; AC5, type 5 adenylyl cyclase; SERCA, sarco/endoplastic reticulumn calcium ATPase; SR, sarcoplasmic reticulumn; RyR2, ryanodine receptor 2.

AngII in turn is involved in the activation of several signaling pathways, including mitogen-activated protein kinase (MAPK) and ROS production, leading to cardiac remodeling and dysfunction^61^. In this study, we confirmed that phosphorylation of p38 MAPK and expression of NOX4 and XO were significantly increased in the BO group and these increases were significantly decreased by Cap. These findings suggest that activation of MAPK signaling and increased expression of ROS-producing enzymes might augment occlusal disharmony-induced cardiac dysfunction in association with AT1R-mediated PKCδ phosphorylation (Fig. 8).

Overall, our results, together with previous findings, suggest that the angiotensin converting enzyme inhibitor Cap might broadly inhibit stress-induced cardiomyopathy, which in turn might lead to improved longevity and reduced physiological frailty.

## Limitations

Patients with occlusal disharmony, which derives from various conditions associated with poor oral health, have not only psychological stress, but also a higher incidence of comorbidies, including cardiovascular disease, although the causal mechanism is not yet clear^62,63^. As regards animal models, BO mice, which have previously been used in research on occlusal disharmony, have increased plasma corticosterone levels, a marker of psychological stress^18,22,51^, suggesting that the BO mouse model might be representative of the comorbidies associated with oral frailty in humans. Notably, this model has some limitations because the previuouly reported BO mouse model was established within a 2-week experimental period^18,22,51^, and thus might reflect only the initial stage of the comorbidies, rather than advanced lesions.

Pitt et al. demonstrated in the Randomized Aldactone Evaluation Study (RALES) that administration of ACE inhibitors reduced morbidity and mortality rates more in patients with severe heart failure if combined with the aldosterone antagonist spironolactone^64^. These findings indicate that physiologically active levels of aldosterone persist even during long-term inhibition of RAS with ACE inhibitor or ARB; this is called aldosterone breakthrough. Selective antagonism of AT1R is known to be associated with a substantial increase of serum Ang II level^65^. It is therefore suggested that increased serum Ang II levels may stimulate aldosterone secretion through AT2R when AT1R is blocked with ARB^66,67^. Naruse et al.^68^ reported that plasma aldosterone concentration was significantly decreased during the first 4 weeks of ARB administration, but aldosterone breakthrough occurred after 8 weeks with an increase of serum Ang II concentration in stroke-prone spontaneously hypertensive rats. Hashimoto et al.^69^. reported that urinary aldosterone excretion was significantly decreased by 4 weeks of treatment with ARB, but aldosterone breakthrough occurred after 12 weeks of ARB administration in Tsukuba hypertensive mice These data suggest that longer-term follow-up using a chronically psychologically stressed model may provide additional information on the development of comorbidities induced by oral frailty.

## Methods

### Mice and experimental protocols

All experiments were performed on male 16-week-old C57BL/6 mice obtained from CLEA Japan (Tokyo, Japan). Mice were group-housed at 23 °C under a 12–12 light/dark cycle with lights on 8:00 AM in accordance with the standard conditions for mouse studies by our group^18–20,70^. Both food and water were available ad libitum. Occlusal disharmony in mice was induced by introducing a 0.7-mm BO, by cementing a suitable applicance onto the mandibular incisor under anesthesia with intraperitoneal medetromidine (0.03 mg/mL), midazolam (0.5 mg/mL), and butorphanol (0.5 mg/mL)^18–20,22^ (Fig. 1A). Mice were group-housed (approximately 3 mice per cage) and were divided into four groups: a normal control group (Control), a BO-only treatment group (BO), a Cap (#C8856; Sigma-Aldrich, St. Louis, MO, USA)-only treatment group (Cap), and a BO plus Cap treatment group (BO + Cap) (Fig. 1B).

In order to examine the effects of ARB on BO-mediated cardiac dysfunction, we performed the same experiments using losartan (#PHR1602; Sigma-Aldrich) instead of Cap. Cap^71,72^ and losartan^73^ were directly dissolved in drinking water (0.1 mg/mL; freshly prepared every day). Because the BO mice cannot easily eat the standard pellet food (CE-2: 334.9 kcal/100 g; CLEA Japan), but can take paste food, the standard pellet food was changed to paste food 3 days before the BO treatment in all groups, as in previous studies^18,22^. Body weight (BW), food intake, and water intake were monitored throughout the 2-week experimental period (Control: *n* = 6, BO:* n* = 6, Cap: *n* = 6, BO + Cap: *n* = 6).

### Ethical approval

All animal experiments complied with the ARRIVE guidelines^74^ and were carried out in accordance with the National Institutes of Health guide for the care and use of laboratory animals^75^ and institutional guidelines. The experimental protocol was approved by the Animal Care and Use Committee of Tsurumi University (No. 29A041).

### Serum corticosterone and Ang II measurements

The serum was separated from blood samples collected from the heart of the control, BO, Cap and BO + Cap groups (*n* = 6 each) under anesthesia at 14 days after the BO treatment. Blood sampling was done in the morning (9:00–10:00 AM) and the procedure was completed within 30 s from the time of contact with the mouse^76^. The separated serum samples were frozen at -80 °C until measurement. The serum corticosterone levels were determined using a Corticosterone HS EIA kit (#AC-15F1; Immunodiagnostic System Ltd., Tyne & Wear, UK) in accordance with the manufacturer’s instructions^18,22^. Serum Ang II levels were determined using an angiotensin II ELISA Kits (#ADI-900-204; Enzo Life Science, Farmingdale, NY, USA).

### Physiological experiments

Mice (Control: *n* = 6, BO: *n* = 6 Cap: *n* = 6, BO + Cap: *n* = 6) were anesthetized via a mask with isoflurane vapor (1.0–1.5% v/v) titrated to maintain the lightest possible anesthesia, and echocardiographic measurements were performed by means of ultrasonography (TUS-A300, Toshiba, Tokyo, Japan) at 14 days after BO treatment^15,16^.

All LV dimensions are presented as the average of four consecutive selected beats. Heart rate (HR) was determined from the cardiac cycles recorded on the M-mode tracing, using at least three consecutive beats. The other parameters were calculated from M-mode-derived LV dimensions using the Teichholz formula^77^:

- $$ \begin{aligned} {\text{EDV}}\, & = \,({7}\, \times \,{\text{LVIDd}}^{{3}} /{1}000)/\left( {{2}.{4}\, + \,\left( {{\text{LVIDd}}/{1}0} \right)} \right) \, \;{\text{and }} \\ {\text{ESV}} & \, = \,({7}\, \times \,{\text{LVIDs}}^{{3}} /{1}000)/\left( {{2}.{4}\, + \,\left( {{\text{LVIDd}}/{1}0} \right)} \right) \, ({\text{mL}}). \\ \end{aligned} $$
  LVEDV (mL): left ventricular end-diastolic volume.
  LVESV (mL): left ventricular end-systolic volume.
  LVIDd (mm): left ventricular internal dimension at end-diastole.
  LVIDs (mm): left ventricular internal dimension at end-systole.
- $$ {\text{Stroke }}\;{\text{volume}}\; \, \left( {{\text{SV}}} \right)\, = \,{\text{EDV}} - {\text{ ESV }}({\text{mL}}) $$
- $$ {\text{Cardiac}}\;{\text{ output }}\left( {{\text{CO}}} \right)\, = \,{\text{HR }} \times {\text{ SV }}({\text{mL}}/{\text{min}}) $$
- $$ {\text{Left}}\;{\text{ ventricular}}\;{\text{ ejection}}\;{\text{ fraction}}\; \, \left( {{\text{EF}}} \right)\, = \,{1}00\, \times \,{\text{SV}}/{\text{EDV }}(\% ) $$
- $$ {\text{Left }}\;{\text{ventricular}}\;{\text{ fractional}}\;{\text{ shortening}}\; \, \left( {\% {\text{FS}}} \right)\, = \,{1}00 \, \times \, \left( {{\text{LVIDd}} - {\text{LVIDs}}} \right)/{\text{LVIDd }}(\% ) $$

All LV dimensions calculated using the Teichholz formula in wild-type control mice (12-week-old C57BL/6 mice) shown in this study were consistent with those reported in previous studies by us^70^ and another group^78^.

After the completion of echocardiographic measurements, mice were anesthetized with isoflurane (1.0–1.5% v/v) via a mask at room temperature and killed by cervical dislocation. The heart were quickly removed^79^, weighed, then immediately frozen in liquid nitrogen with Tissue-Tek OCT compound (Sakura Finetek, Torrance, CA, USA), and stored until sectioning.

### Myocyte cross-sectional area

Cross sections (10 μm) were cut with a cryostat (CM1900, Leica Microsystems, Nussloch, Germany) at − 20 °C. The sections were air-dried and fixed with 4% paraformaldehyde (v/v) in 0.1 M phosphate-buffered saline (pH 7.5). The section were then stained with hematoxyline and eosine (HE) and observed under a light microscope (BX61, Olympus Co., Tokyo, Japan). Micrographs were taken with a digital camera (DP-72, Olympus Co.) connected to a personal computer. The cross-sectional size of muscle fibers was evaluated by measuring the cross-sectional area (CSA). The CSA of 20–50 myocytes was measured with image analysis software (Image J 1.45) and averaged the mean value in each mouse^80^.

### Evaluation of fibrosis and apoptosis

We employed Masson-trichrome staining using the Accustain Trichrome Stain kit (#HT15-1KT; Sigma-Aldrich, St. Louis, MO, USA) in accordance with the manufacturer’s protocol^19^. In Masson staining, fibers stained in aniline blue were collagen fibers, while those stained in red were muscle fibers. Sections of the cardiac tissues were outlined manually to define regions of interest (ROIs) (Control: *n* = 6, BO: *n* = 6, Cap: *n* = 6, BO + Cap: *n* = 6). We measured the percentage fibrosis within 3–5 ROIs for each section, using Image J 1.48v software (National Institute of Health, Bethesda, MD, USA https://imagej.nih.gov/ij/download.html)^80^.

DNA fragmentation was determined by TUNEL staining using an Apoptosis in situ Detection kit (#293-71501; FUJIFILM Wako Pure Chemical Corporation, Osaka, Japan). The total number of TUNEL-positive nuclei was counted manually in six sections in the four groups (Control: *n* = 4, BO: *n* = 4, Cap: *n* = 4, BO + Cap: *n* = 4).

### Western blotting

Cardiac tissue excised from the mice (Control: *n* = 6, BO: *n* = 6, Cap: *n* = 6, BO + Cap: *n* = 6) was homogenized in a Polytron (Kinematica AG, Lucerne, Switzerland) in ice-cold RIPA buffer (#89900; Thermo Fisher Scientific, Waltham, MA, USA: 25 mM Tris–HCl (pH 7.6), 150 mM NaCl, 1% sodium deoxycholate, 0.1% SDS) with addition of Halt™ Protease Inhibitor Cocktail, EDTA-free (#87785; Thermo Fisher Scientific) and the homogenate was centrifuged at 13,000×*g* for 10 min at 4 °C. The supernatant was collected and the protein concentration was measured using a DC protein assay kit (Bio-Rad, Hercules, CA, USA). Equal amounts of protein (5 μg) were subjected to 12.5% SDS–polyacrylamide gel electrophoresis and blotted onto PVDF membrane (#IPVH00010; Millipore, Burlington, MA, USA).

Western blotting was conducted with commercially available antibodies^13–16^. Primary antibodies against α-SMA (1:1000, #19245), p38 (1:1000, #8690), phospho-p38 (1:1000, Thr-180/Tyr-182, #4511), PKC (1:1000, #2058), phospho-PKCδ (1:1000, Thy505, #9374), phospho-PKCδ (1:1000, Tyr311, #2055), ASK1 (1:1000, #8662), phospho-ASK1 (1:1000, Thr-845, #3765), CaMKII (1:1000, #3362), phospho-CaMKII (1:1000, Thr286, #3361), and Bax (1:1000, #2775) were purchased from Cell Signaling Technology (Boston, MA, USA), primary antibody against glyceraldehyde-3-phosphate dehydrogenase (GAPDH) (1:200, sc-25778) was purchased from Santa Cruz Biotechnology (Santa Cruz, CA, USA) and primary antibodies against PLB (1:5000, #A010-14), phospho-PLB (1:5000, Thr-17, #A010-13; 1:5000, Ser-16, A010-12), phospho-RyR2 (1:2000, Ser-2808, #A010-30) and phospho-RyR2 (1:2000, Ser-2814, #A010-31) were purchased from Badrilla (Leeds, UK). RyR2 (1:1000, #MA3-916) was purchased from Thermo Fisher (Rockland, IL, USA). Primary antibodies against NOX4 (1:1000, #ab133303) and XO (1:1000, #ab109235) were purchased from Abcam (Cambridge, UK) and primary antibody against oxidized CaMKII (Met-281/282) (1:1000, #07-1387) and collagen type 1 (1:1000, AB765P) were purchased from Millipore (Billerica, MA, USA). Primary antibody against collagen type 3 (1:1000, NB600-594) was purchased from Novus Biologicals (Centennials, CO, USA). The primary and secondary antibodies were diluted in Tris-buffered saline (pH 7.6) with 0.1% Tween 20 and 5% bovine serum albumin. The blots were visualized with enhanced chemiluminescence solution (ECL: Prime Western Blotting Detection Reagent, GE Healthcare, Piscataway, NJ, USA) and scanned with a densitometer (LAS-1000, Fuji Photo Film, Tokyo, Japan). The amount of expression in the Control was taken as 100% in each determination in accordance with previous studies^15,16^.

The reason why there are different numbers of samples in different western blotting figures (Figs. 2B, 3C-E, 4C, 5C, D, 6A–F, 7A–D) is that we excluded outliers (extremely low or high values, compared to others in the same group).

### Immunostaining

Oxidative DNA damage in the myocardium was evaluated by immunostaining for 8-OHdG using the Vector M.O.M. Immunodetection system (#PK-2200, Vector Laboratories, Inc. Burlingame, CA, USA)^18,19,81,82^. Cross sections (Control: *n* = 5, BO: *n* = 5, Cap: *n* = 5, BO + Cap: *n* = 5) were cut with a cryostat at − 20 °C at 10 μm, air-dried and fixed with 4% paraformaldehyde (v/v) in tris-buffered saline (TBS)-T for 5 min at room temperature. Antigen retrieval was achieved with 0.1% citrate plus 1% Triton X-100 for 30 min at room temperature, then the sections were washed with TBS-T, incubated with 0.3% horse serum in TBS-T for 1 h at room temperature, and blocked with M.O.M. blocking reagents (Vector Laboratories, Burlingame, CA, USA) overnight at 4 °C. For the positive control, sections were incubated with 0.3% H_2_O_2_ in TBS-T before the anti-8-OHdG antibody treatment. The sections were incubated with anti-8-OHdG antibody (8.3 μg/mL in M.O.M. Dilute; clone N45.1 monoclonal antibody; Japan Institute for the Control of Aging, Shizuoka, Japan) overnight at 4 °C in a humidified chamber, and then incubated with 0.3% H_2_O_2_ in 0.3% horse serum for 1 h at room temperature to inactivate endogenous peroxidase, rinsed with TBS-T, incubated with anti-horse IgG in M.O.M. Dilute, and processed with an ABC kit (Vector Laboratories, Inc. Burlingame, CA, USA). We calculated the ratio of 8-OHdG nuclei with oxidative DNA damage (stained dark blown) per total cell number.

### Statistical analysis

Data are presented as means ± standard deviation (SD). Comparisons were performed using two-way repeated-measures ANOVA followed by the Bonferroni post hoc test (Fig. 1B, Supplementary Fig. 1A, B) or one-way ANOVA followed by Tukey’s post hoc test (Figs. 1C, 2A–C, E, F, 3B–E,  4B, C, 5B–D, 6A–F and 7A–D). Differences were considered significant when *P* < 0.05.

### Supplementary Information

Supplementary Figures.

## Data Availability

The authors confirm that the data supporting the findings of this study are available within the article and its supplementary materials.

## Abbreviations

RAS: Renin-angiotensin system
ACE: Angiotensin-converting enzyme
Ang II: Angiotensin II
AT1R: Ang II type 1 receptor
AT2R: Ang II type 2 receptor
ROS: Reactive oxygen species
SNS: Sympathetic nervous system
BO: Bite-opening
BW: Body weight
Cap: Captopril
ELISA: Enzyme-linked immunosorbent assay
CSA: Cross-sectional area
ERK: Extracellular signal-regulated kinase
LVEF: Left ventricular ejection fraction
%FS: Fractional shortening
LVESV: Left ventricular end-systolic volume
LVDs: Left ventricular internal dimension at end-systole
ARB: Angiotensin receptor blocker
α-SMA: α-Smooth muscle actin
TdT: Terminal deoxyribonucleotidyl transferase
dUTP: Biotin-16-deoxyuridine triphosphate
TUNEL: Terminal deoxyribonucleotidyl transferase (TdT)-mediated biotin-16-deoxyuridine triphosphate (dUTP) nick-end labeling
Bax: B cell lymphoma 2 associated X
8-OHdG: 8-Hydroxy-2′-deoxyguanosine
MAPK: Mitogen-activated protein kinase
NOX4: Nicotinamide adenine dinucleotide phosphate oxidase 4
XO: Xanthine oxidase
PKC: Protein kinase C
ASK1: Apoptosis signal-regulatory kinase 1
CaMKII: Calmodulin kinase II
PLB: Phospholamban
RyR2: Ryanodine receptor 2
LF/HF: Low frequency/high frequency
nHF: Normalized HF
SDNN: Standard deviation of normal R-R intervals
TAC: Transverse aortic constriction
MAPK: Mitpgen-activated protein kinase
HR: Heart rate
