# Supplementary material for: Effects of the angiotensin-converting enzyme inhibitor captopril on occlusal-disharmony-induced cardiac dysfunction in mice
Source: Sci Rep. 2023 Nov 15;13:19927. doi: 10.1038/s41598-023-43099-6 (PMC10651878; doi:10.1038/s41598-023-43099-6)
Supplement: Supplementary file 1 — Supplementary Figures. [file 41598_2023_43099_MOESM1_ESM.pdf]

Supplementary Figures

Effects of the angiotensin-converting enzyme inhibitor captopril on occlusal-disharmony-induced cardiac dysfunction in mice

Aiko Ito [1], Yoshiki Ohnuki [2], Kenji Suita [2], Ichiro Matsuo [3], Misao Ishikawa [4], Takao Mitsubayashi [2], Yasumasa Mototani [2], Kenichi Kiyomoto [2, 3], Michinori Tsunoda [2, 3], Akinaka Morii [2,3], Megumi Nariyama [5], Yoshio Hayakawa [6], Hiroshi Tomonari [1], Satoshi Okumura [2]

[1] Department of Orthodontics, Tsurumi University School of Dental Medicine, Yokohama 230-8501, Japan
[2] Department of Physiology, Tsurumi University School of Dental Medicine, Yokohama 230-8501, Japan
[3] Department of Periodontology, Tsurumi University School of Dental Medicine, Yokohama 230-8501, Japan
[4] Department of Oral Anatomy, Tsurumi University School of Dental Medicine, Yokohama 230-8501, Japan
[5] Department of Pediatric Dentistry, Tsurumi University School of Dental Medicine, Yokohama 236-8501, Japan
[6] Department of Dental Anesthesiology, Tsurumi University School of Dental Medicine, Yokohama 230-8501, Japan

Supplementary figure 1

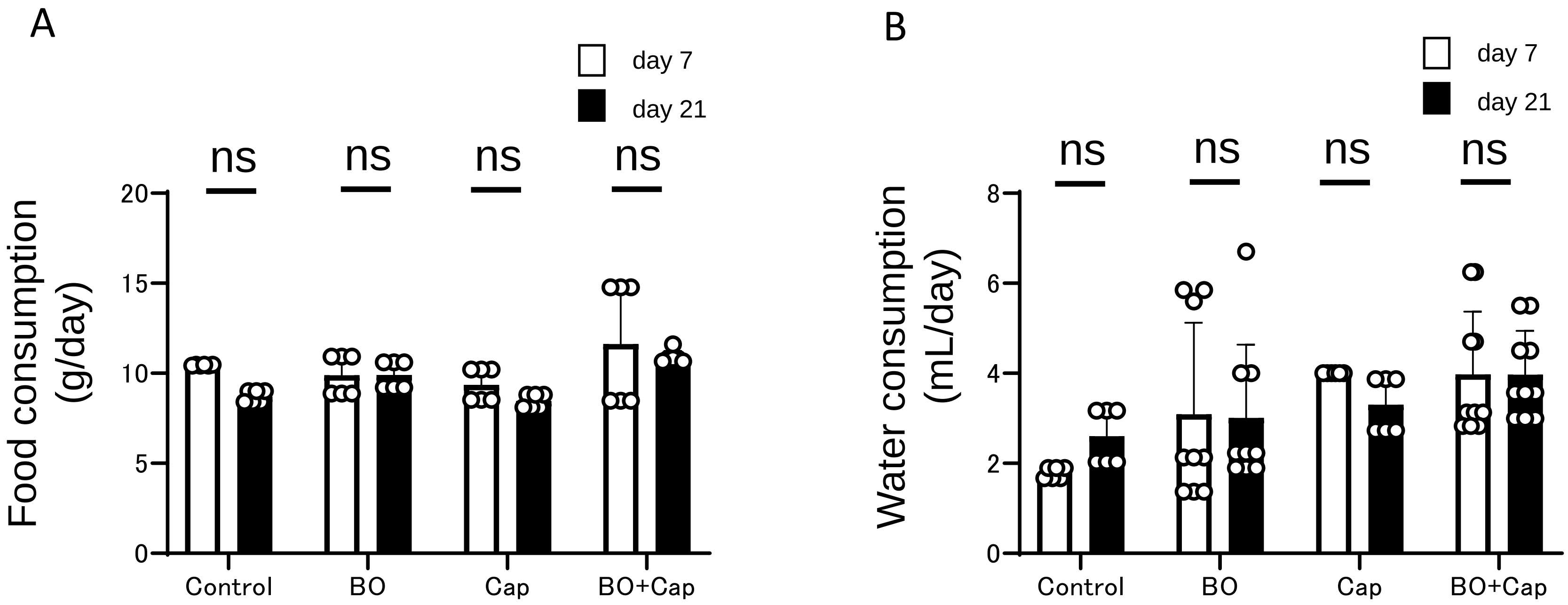

Supplementary Figure 1. Daily consumptions of food and water.
(A, B) No significant difference in daily consumption of food **(A)** or water **(B)** was observed at day 21 (black bars), compared to that at day 7 (white bars), by two-way ANOVA followed by the Bonferroni *post hoc* test.

Supplementary figure 2

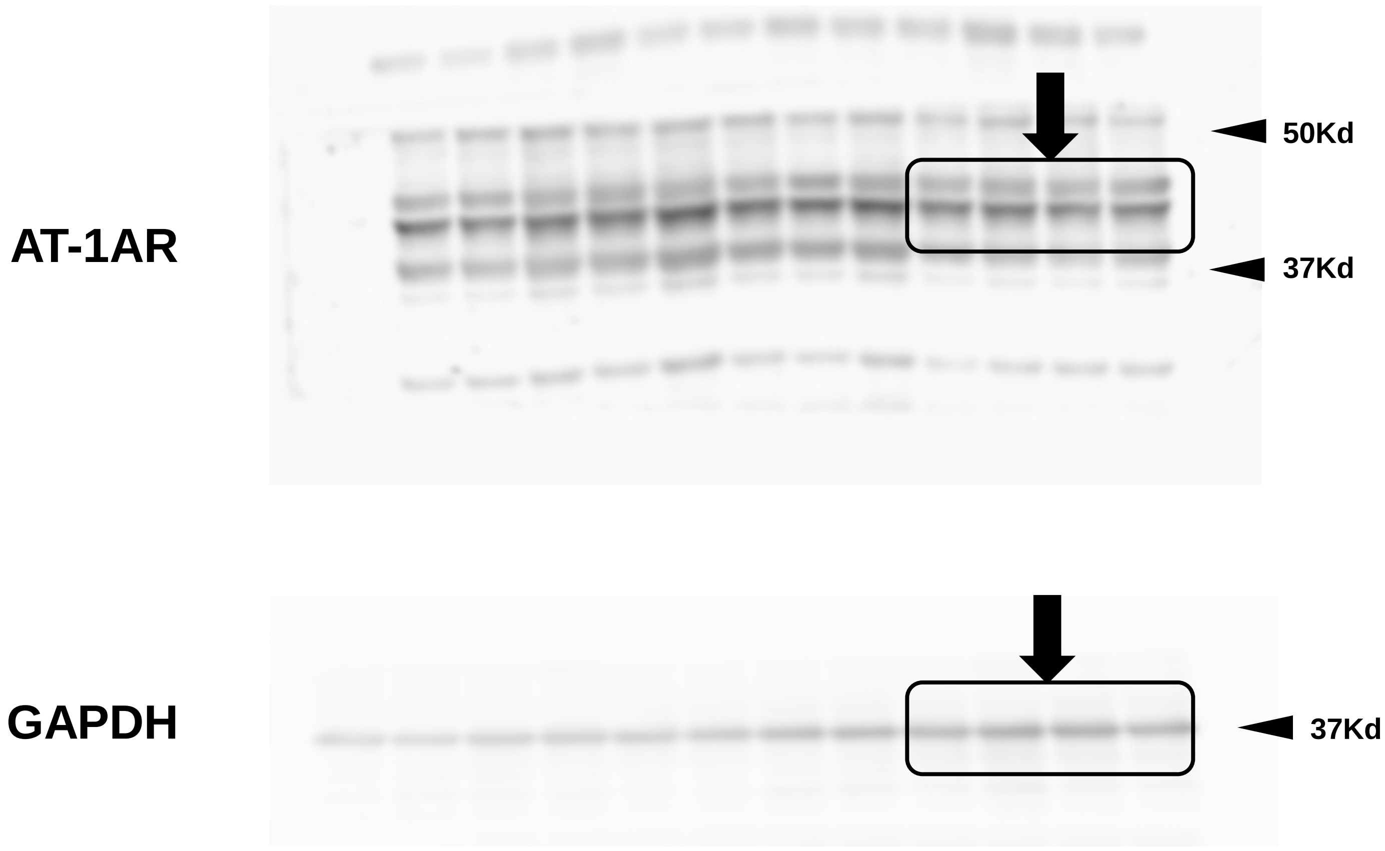

Supplementary Figure 2. Representative full-length western blot.
The amounts of activated AT1-AR (upper panel) and GAPDH (lower panel) are shown. The box outlined in black in each panel, indicated by an arrow, corresponds to the cropped part of the blot shown in **Fig. 2B** in the main article.

Supplementary figure 3

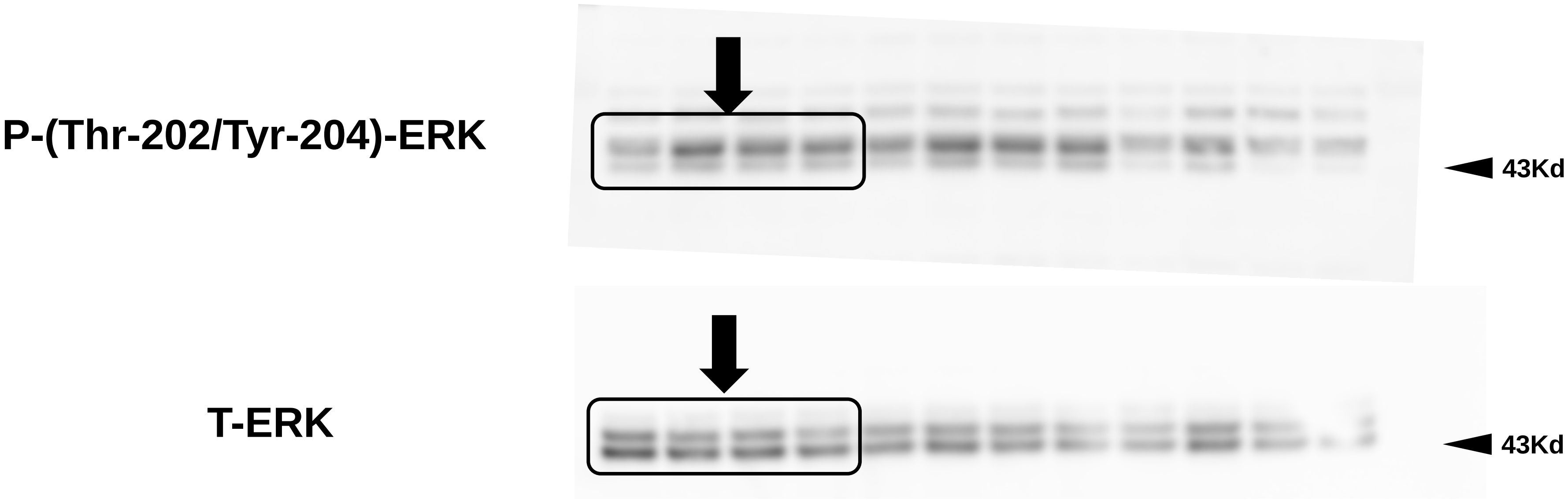

Supplementary Figure 3. Representative full-length western blot.
The amounts of activated P-ERK (upper panel) and T-ERK (lower panel) are shown.
The box outlined in black in each panel, indicated by an arrow, corresponds to the cropped part of the blot shown in **Fig. 2F** in the main article.

Supplementary figure 4

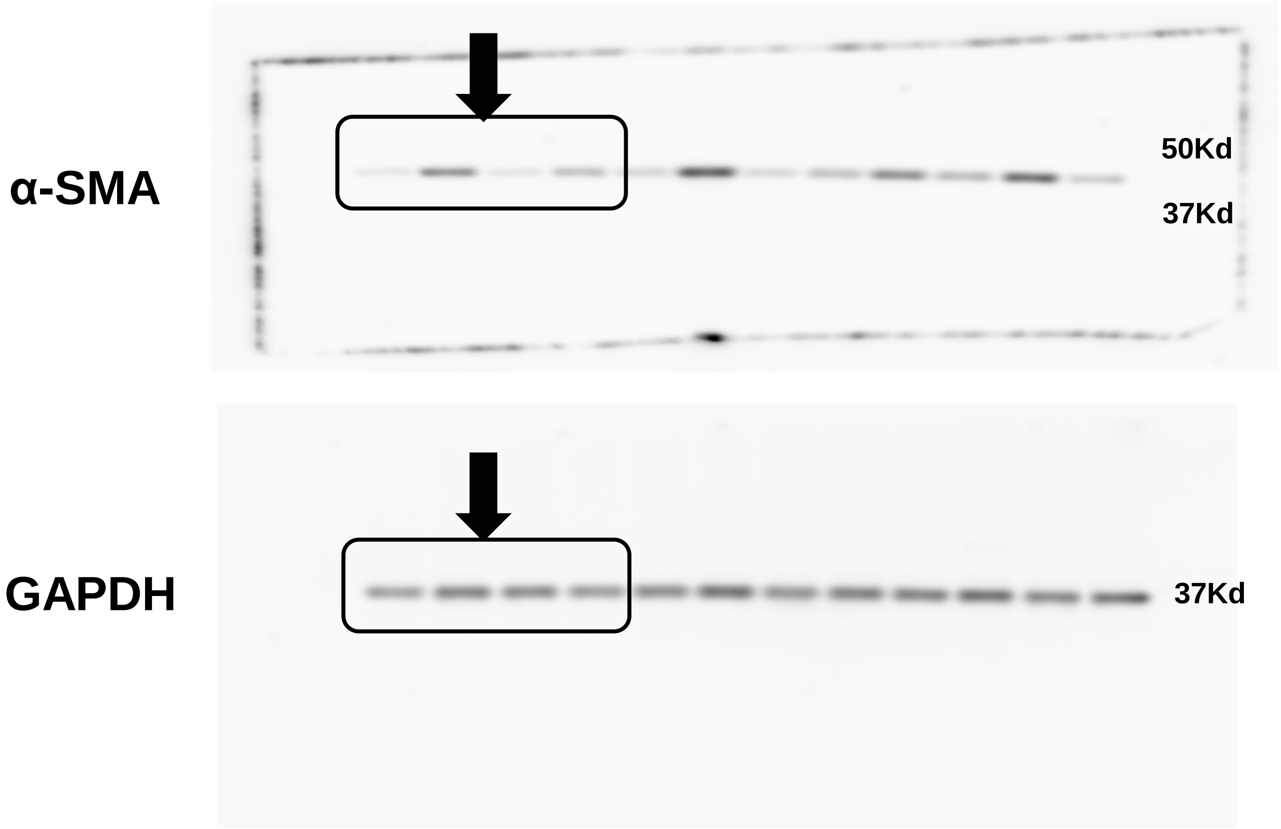

Supplementary Figure 4. Representative full-length western blot.
The amounts of α-SMA (upper panel) and GAPDH (lower panel) are shown. The box outlined in black in each panel, indicated by an arrow, corresponds to the cropped part of the blot shown in **Fig. 3C** in the main article.

Supplementary figure 5

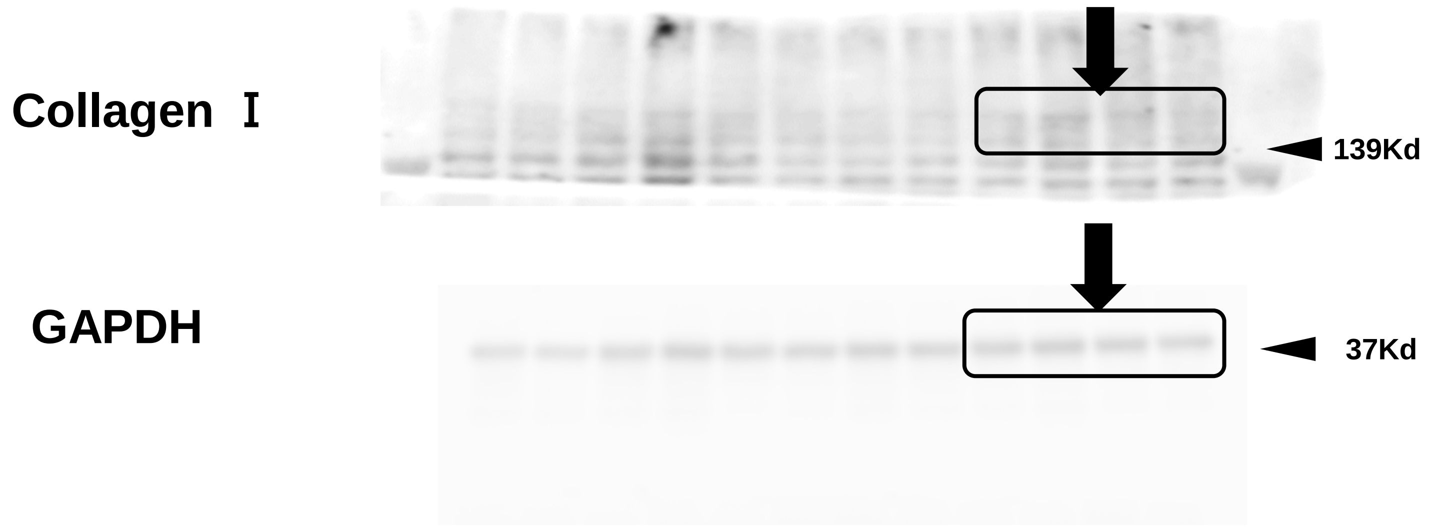

Supplementary Figure 5. Representative full-length western blot.
The amounts of collagen I (upper panel) and GAPDH (lower panel) are shown. The box outlined in black in each panel, indicated by an arrow, corresponds to the cropped part of the blot shown in **Fig. 3D** in the main article.

Supplementary figure 6

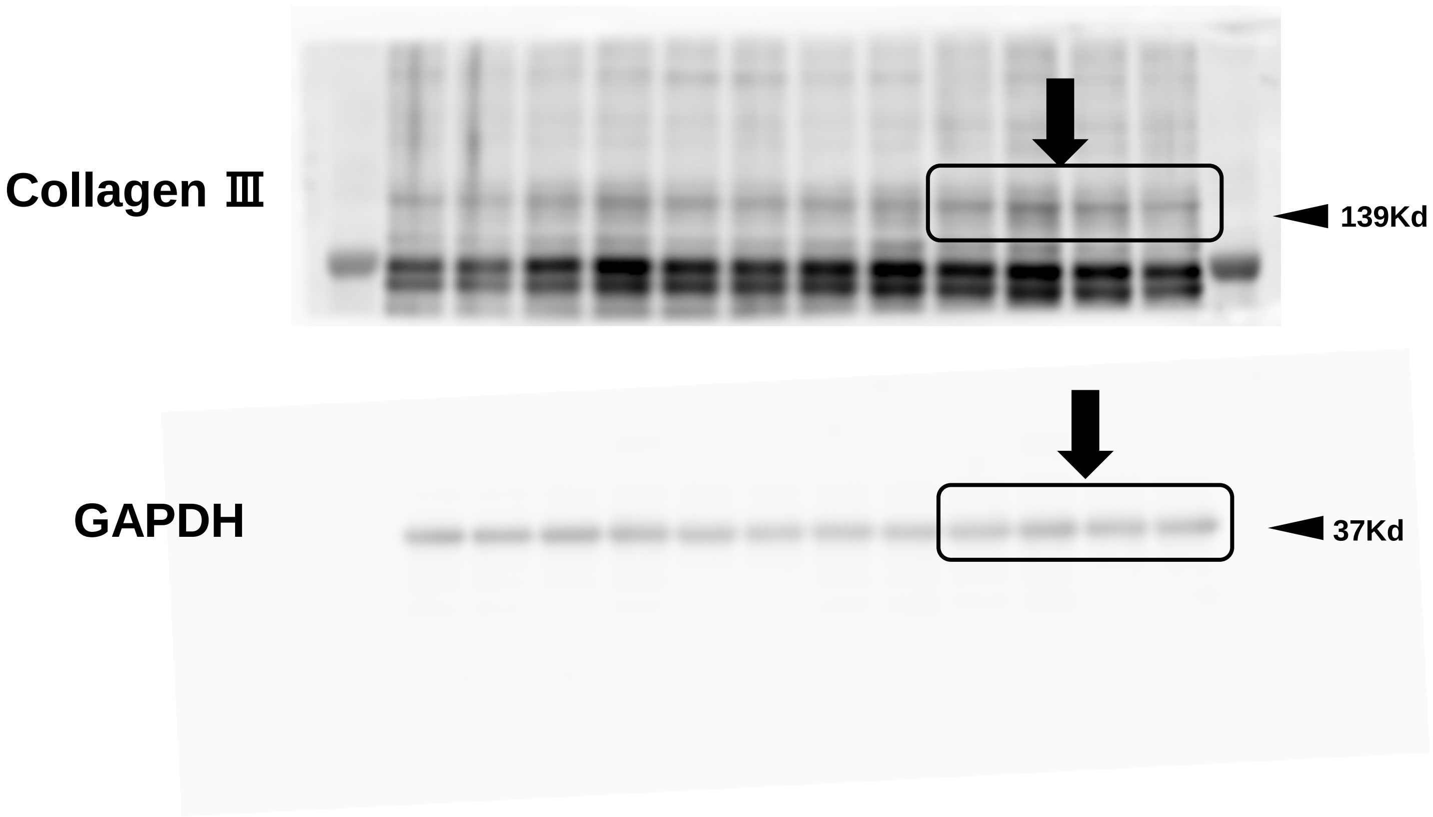

Supplementary Figure 6. Representative full-length western blot.
The amounts of collagen III (upper panel) and GAPDH (lower panel) are shown. The box outlined in black in each panel, indicated by an arrow, corresponds to the cropped part of the blot shown in **Fig. 3E** in the main article.

Supplementary figure 7

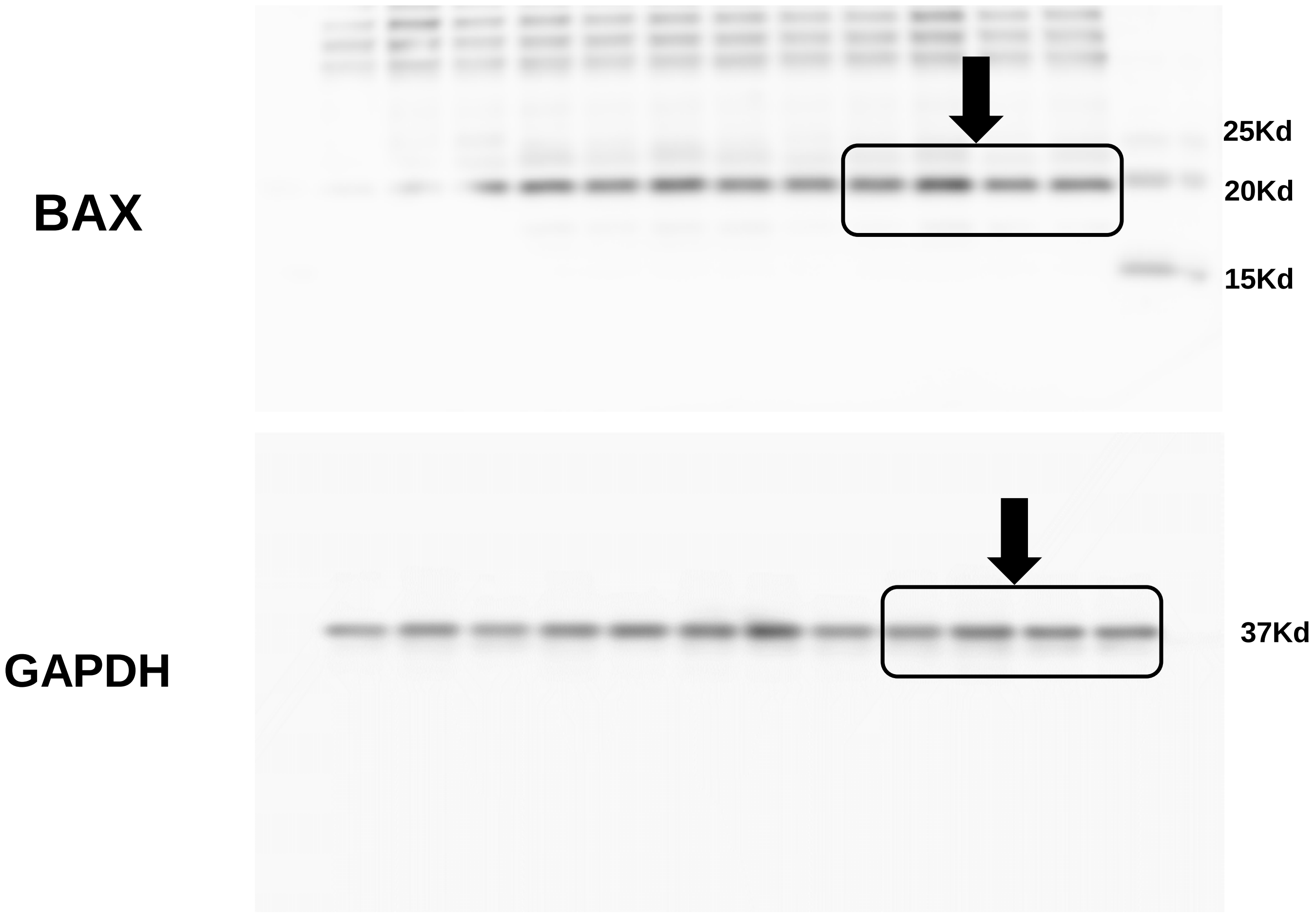

Supplementary Figure 7. Representative full-length western blot.
The amounts of activated BAX (upper panel) and GAPDH (lower panel) are shown.
The box outlined in black in each panel, indicated by an arrow, corresponds to the cropped part of the blot **in Fig. 4C** in the main article.

Supplementary figure 8

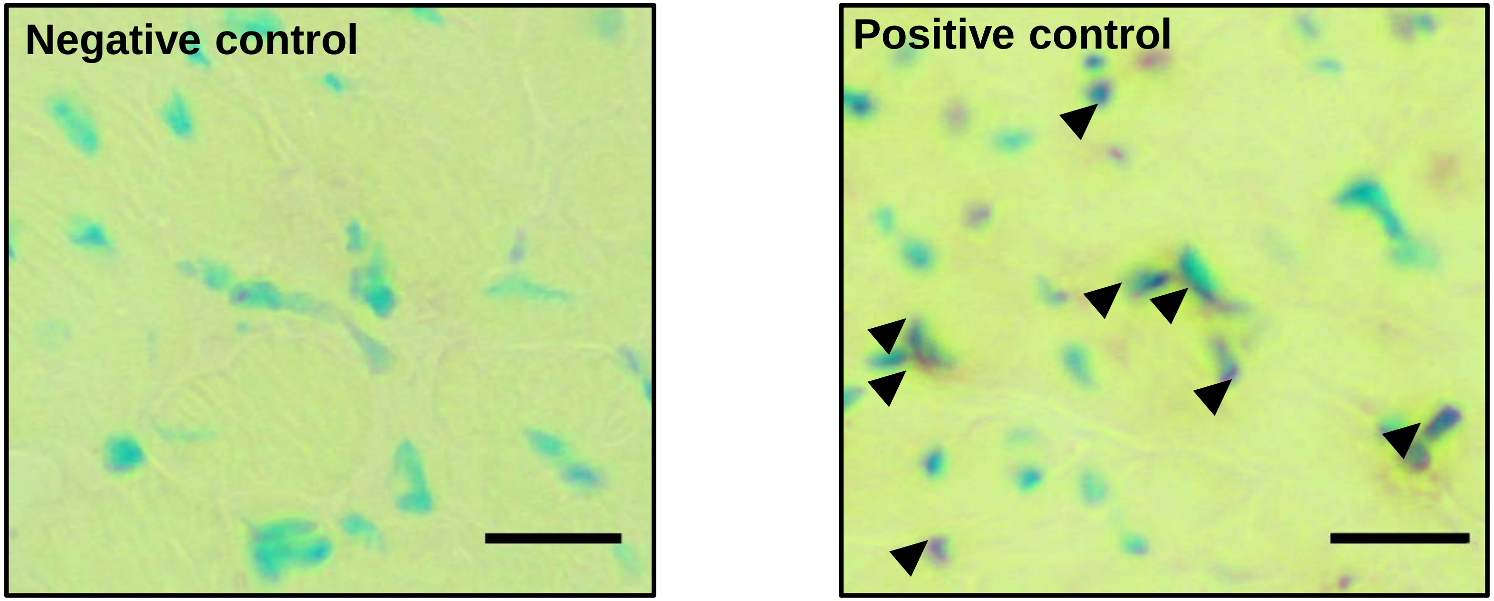

Supplementary Figure 8. Representative images of negative **(A)** and positive **(B)** controls for 8-OHdG immunostaining. Arrowheads indicates positive immunostaining.
Scale bars: 20 μm

Supplementary figure 9

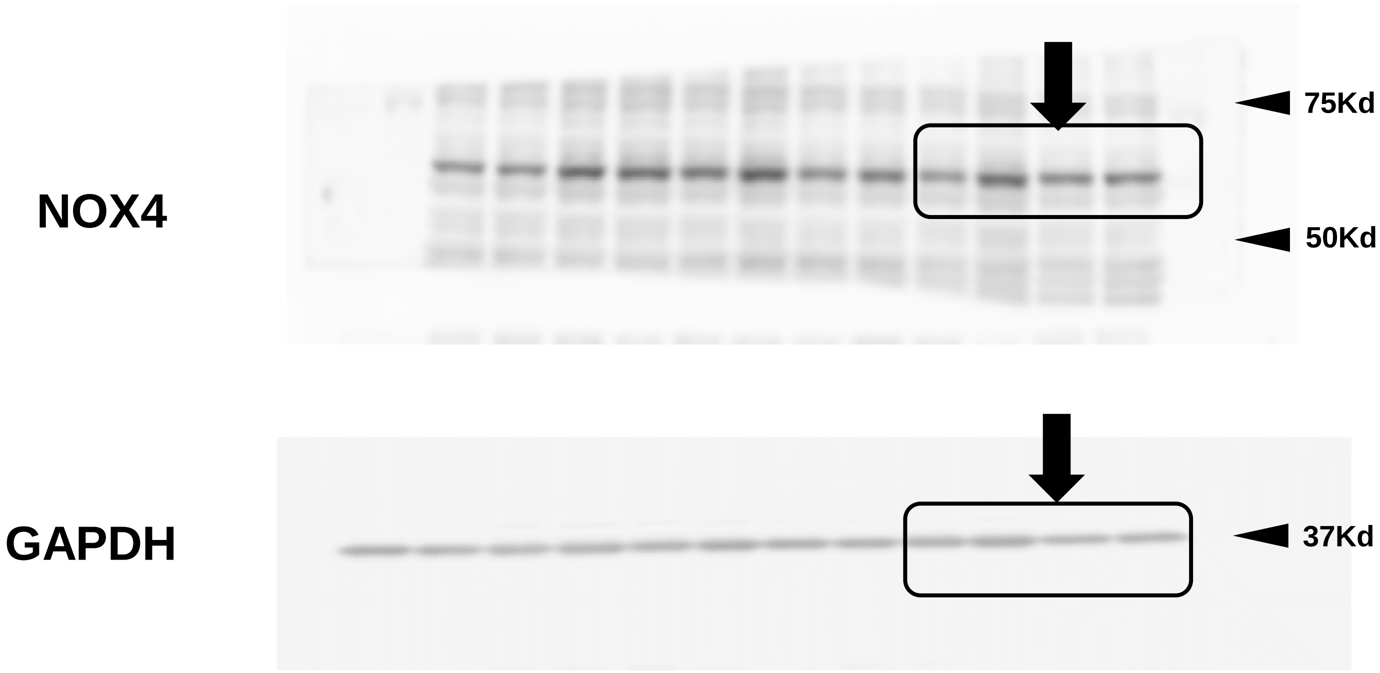

Supplementary Figure 9. Representative full-length western blot.
The amounts of activated NOX4 (upper panel) and GAPDH (lower panel) are shown.
The box outlined in black in each panel, indicated by an arrow, corresponds to the cropped part of the blot shown in **Fig. 5C** in the main article.

Supplementary figure 10

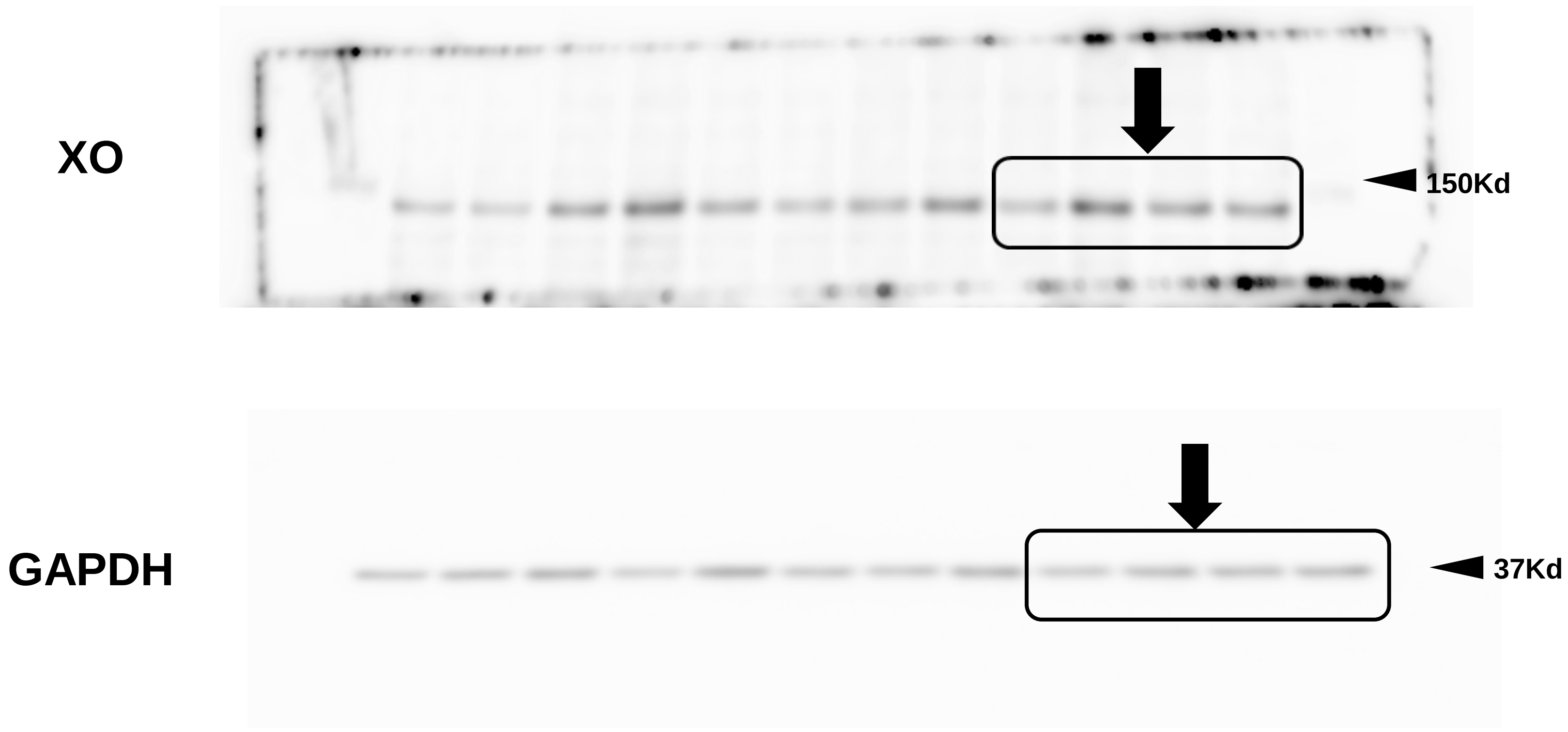

Supplementary Figure 10. Representative full-length western blot.
The amounts of activated XO (upper panel) and GAPDH (lower panel) are shown.
The box outlined in black in each panel, indicated by an arrow, corresponds to the cropped part of the blot shown in **Fig. 5D** in the main article.

Supplementary figure 11

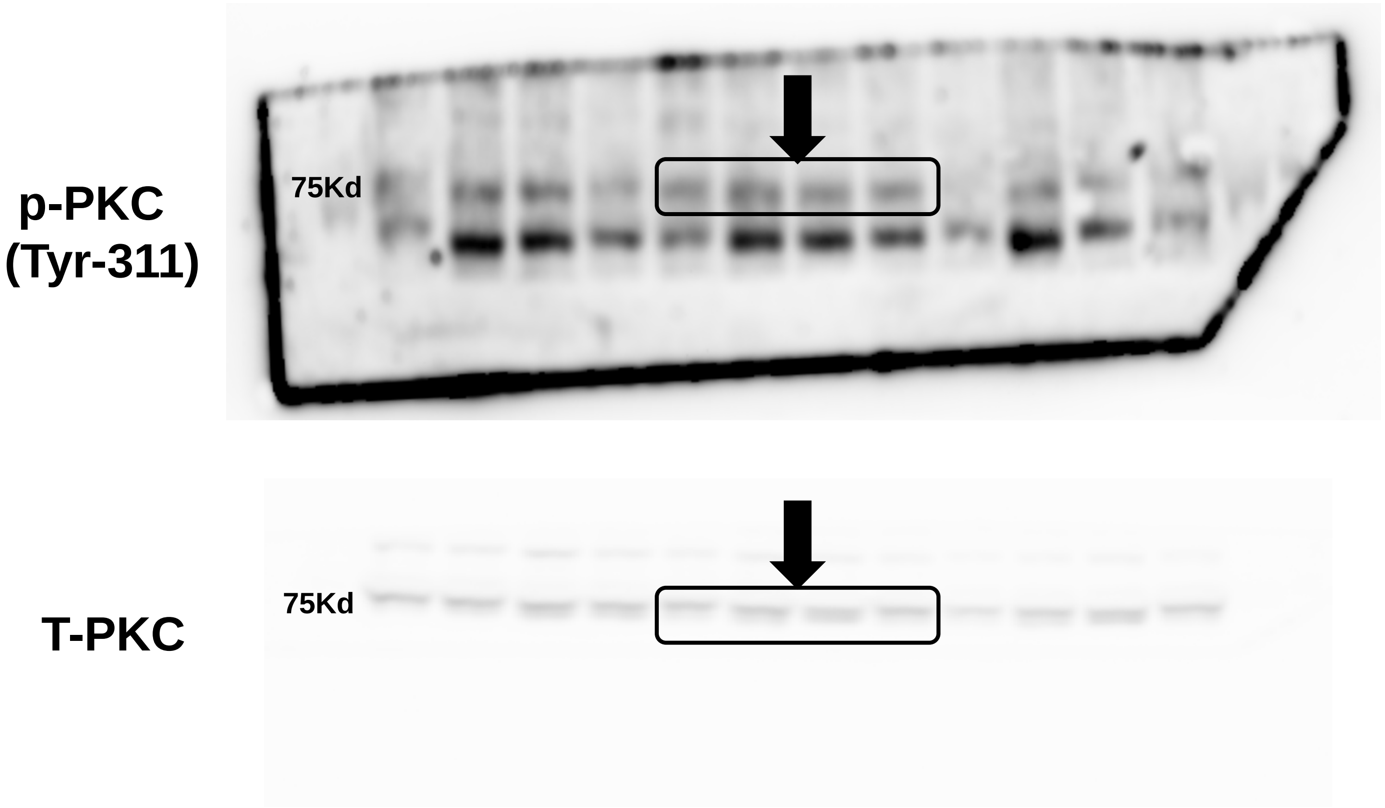

Supplementary Figure 11. Representative full-length western blot.
The amounts of activated P-PKC (Tyr311) (upper panel) and T-PKC (lower panel) are shown. The box outlined in black in each panel, indicated by an arrow, corresponds to the cropped part of the blot shown in **Fig. 6A** in the main article.

Supplementary figure 12

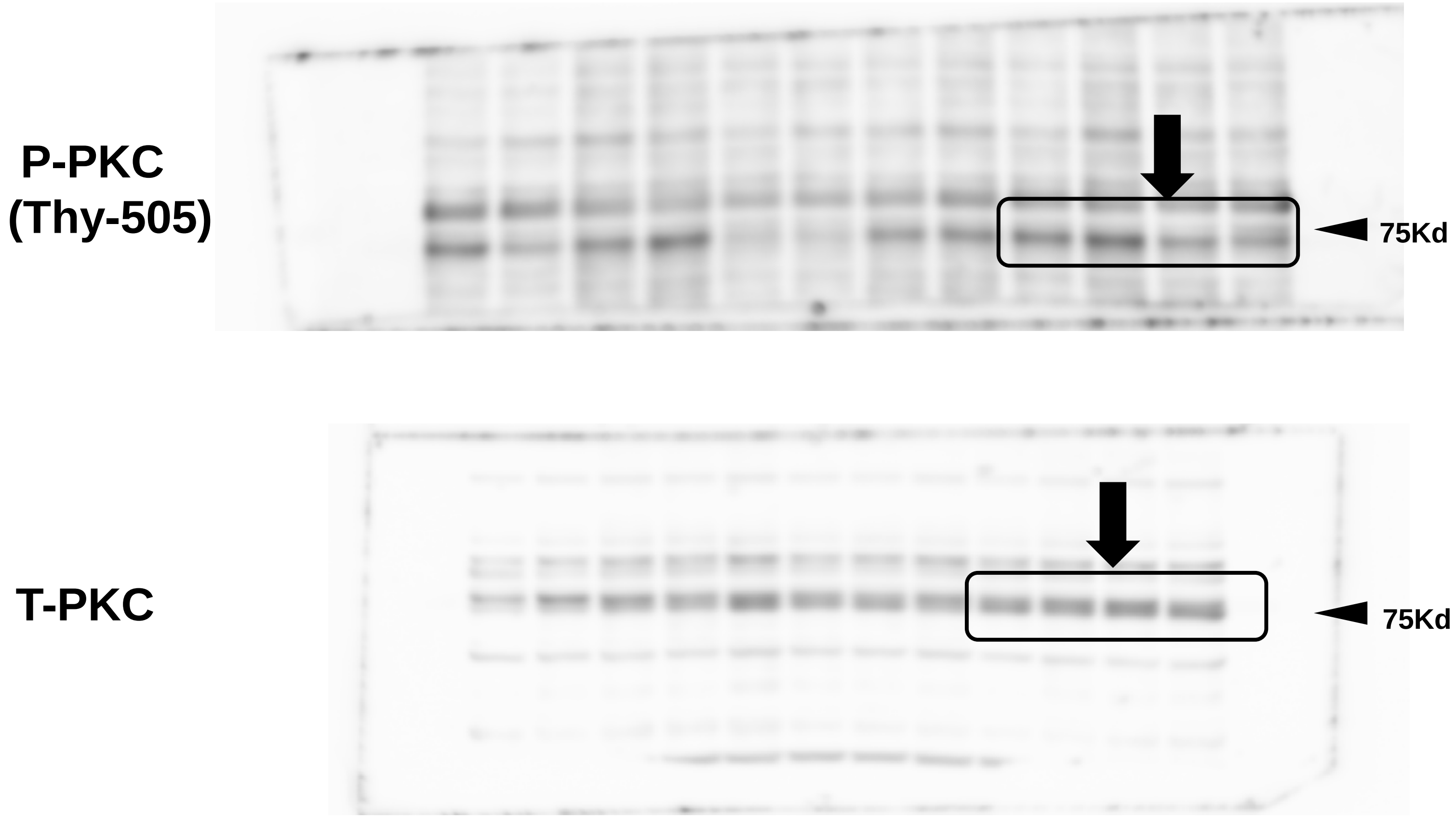

Supplementary Figure 12. Representative full-length western blot.
The amounts of activated P-PKC (Thr-505) (upper panel) and T-PKC (lower panel) are shown. The box outlined in black in each panel, indicated by an arrow, corresponds to the cropped part of the blot shown in **Fig. 6B** in the main article.

Supplementary figure 13

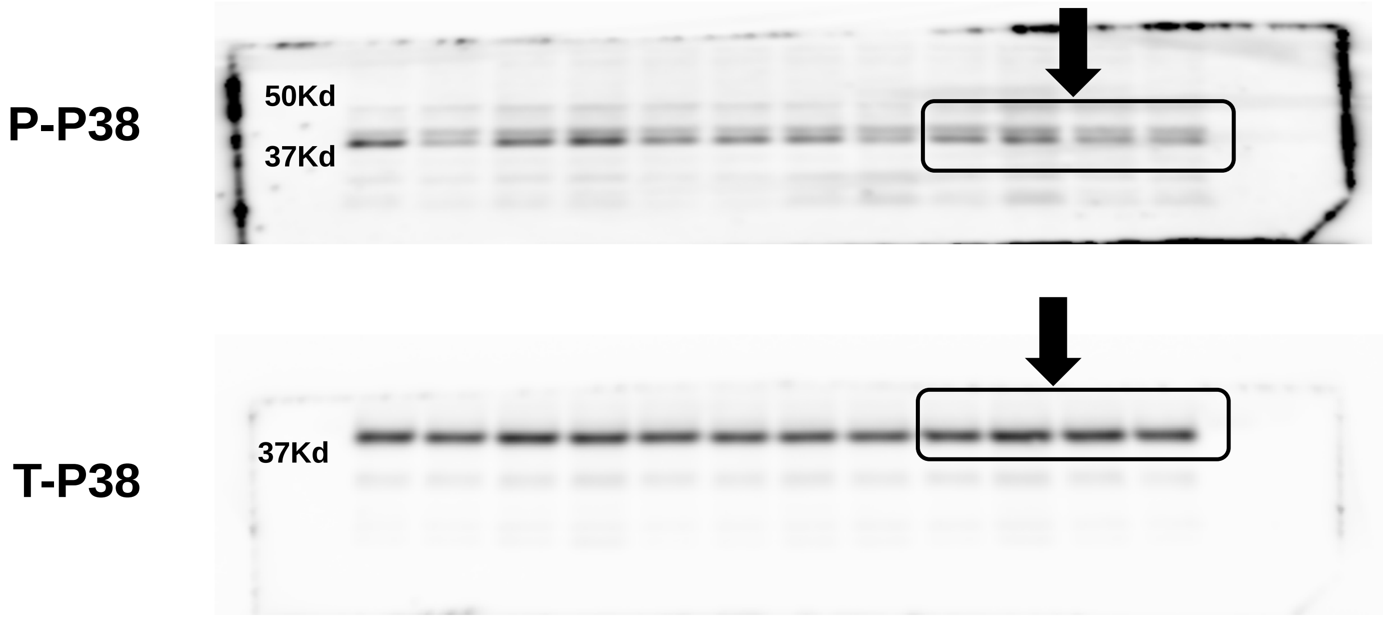

Supplementary Figure 13. Representative full-length western blot.
The amounts of activated P-PKC (Thr-505) (upper panel) and T-PKC (lower panel) are shown. The box outlined in black in each panel, indicated by an arrow, corresponds to the cropped part of the blot shown in **Fig. 6C** in the main article.

Supplementary figure 14

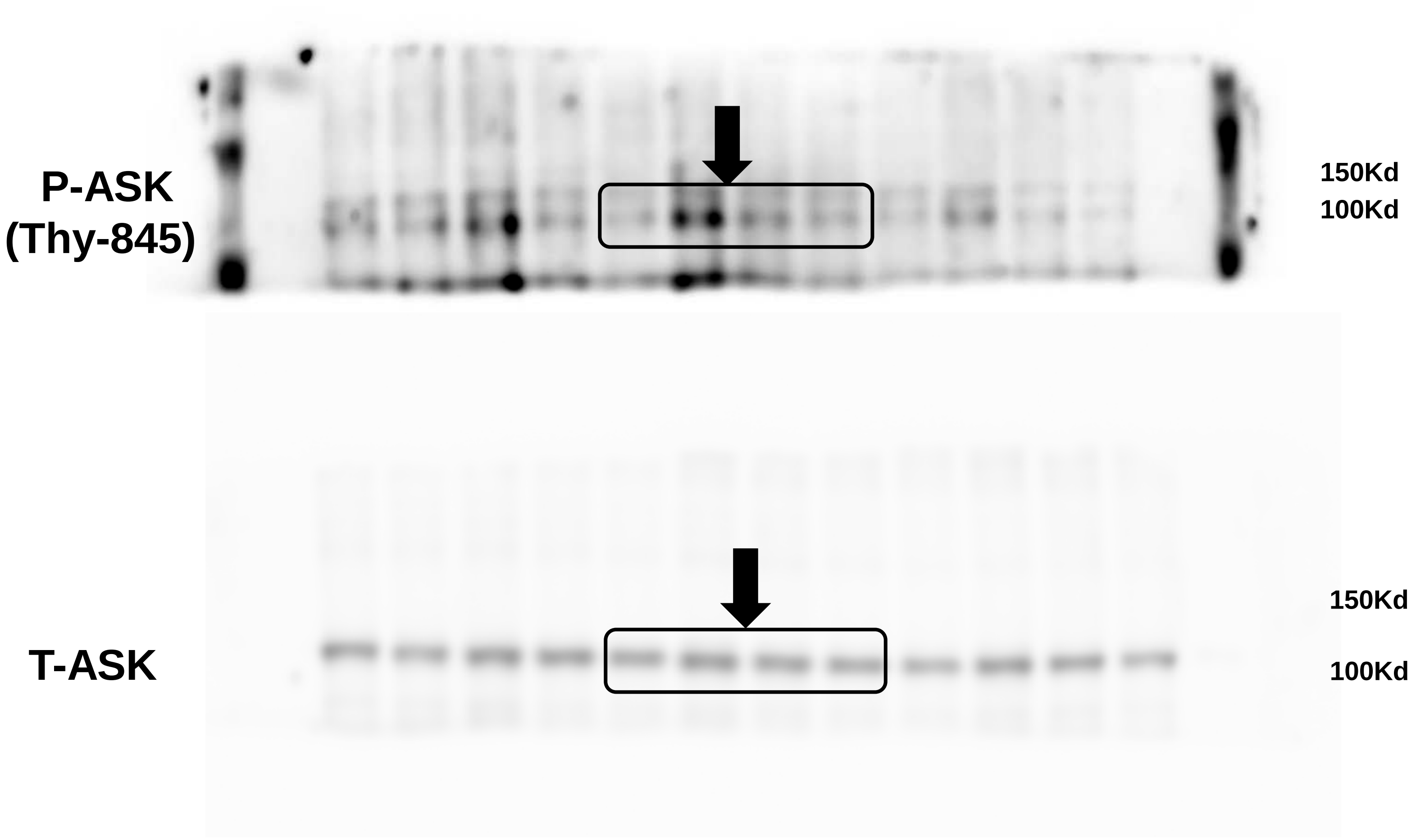

Supplementary Figure 14. Representative full-length western blot.
The amounts of activated P-ASK (Thy-845) (upper panel) and T-ASK (lower panel) are shown. The box outlined in black in each panel, indicated by an arrow, corresponds to the cropped part of the blot shown in **Fig. 6D** the main article.

Supplementary figure 15

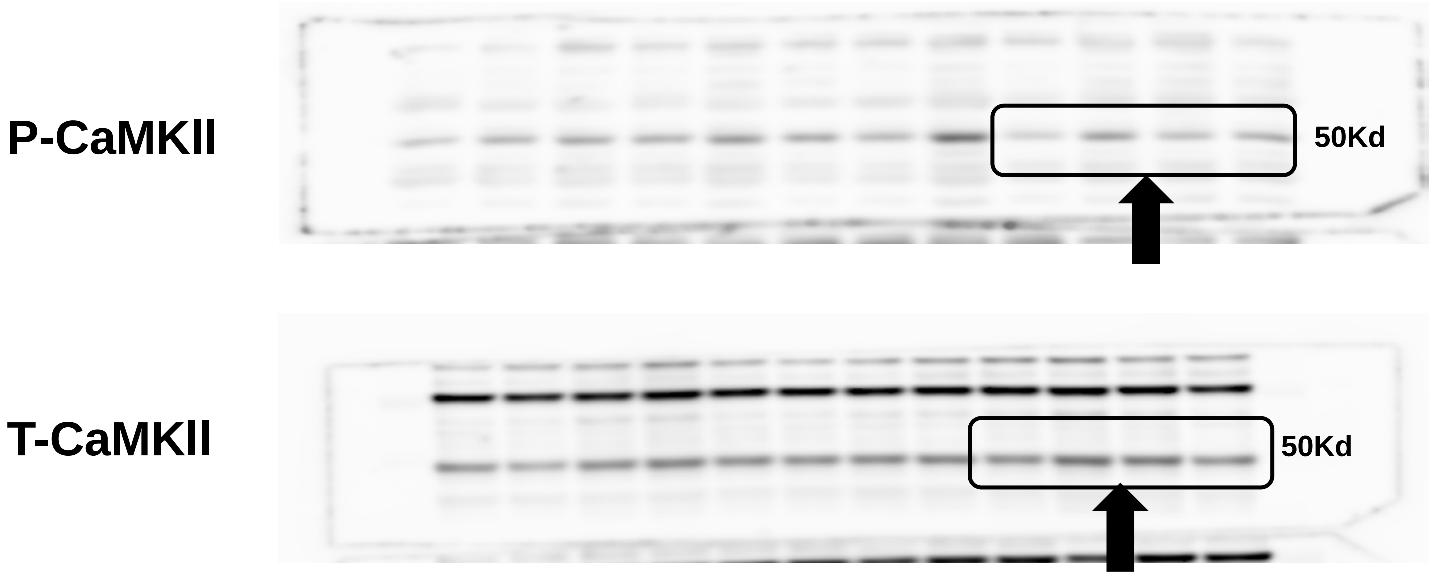

Supplementary Figure 15. Representative full-length western blot.
The amounts of activated P-CaMKll (upper panel) and T-CaMKll (lower panel) are shown. The box outlined in black in each panel, indicated by an arrow, corresponds to the cropped part of the blot shown in **Fig. 6E** in the main article.

Supplementary figure 16

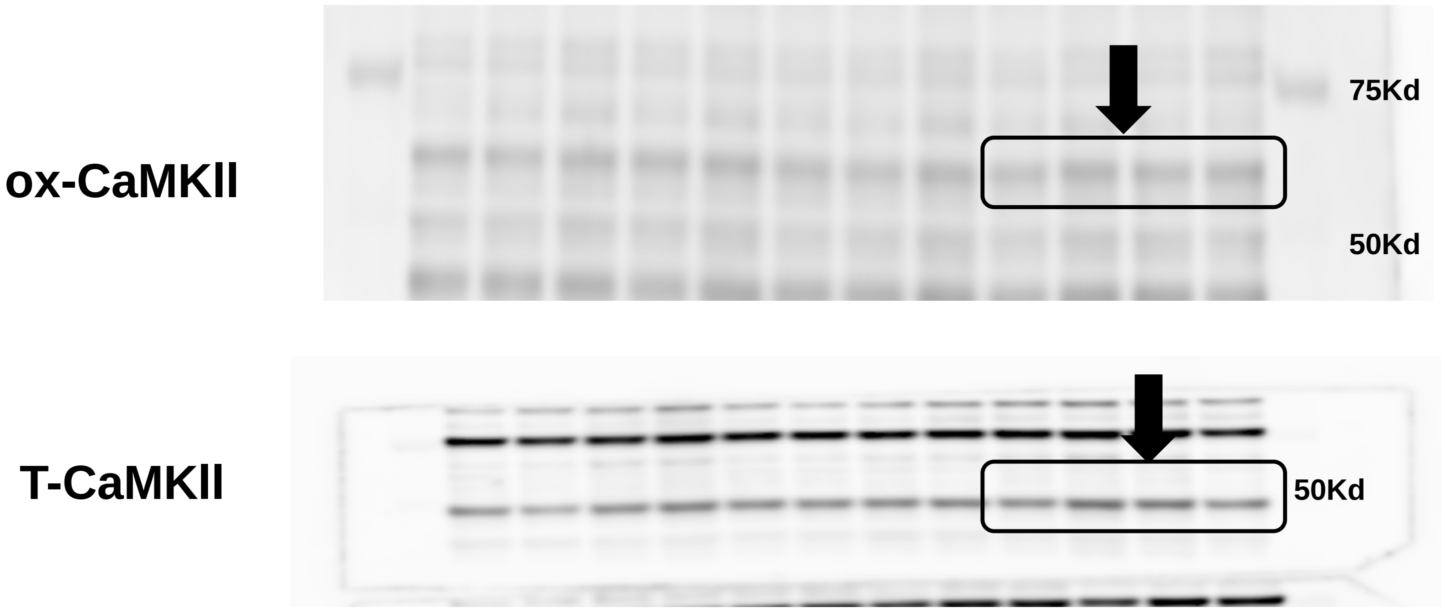

Supplementary Figure 16. Representative full-length western blot.
The amounts of activated ox-CaMKll (upper panel) and T-CaMKll (lower panel) are shown. The box outlined in black in each panel, indicated by an arrow, corresponds to the cropped part of the blot shown in **Fig. 6F** in the main article.

Supplementary figure 17

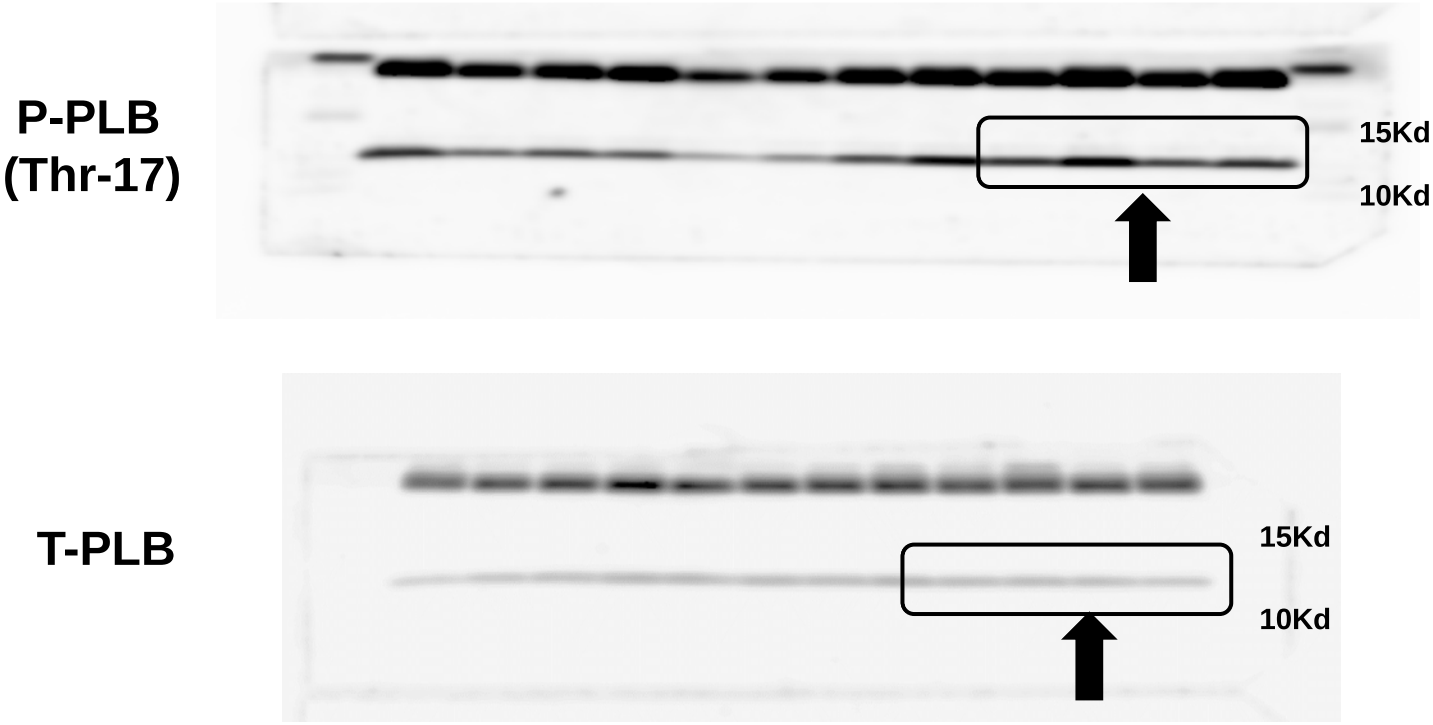

Supplementary Figure 17. Representative full-length western blot.
The amounts of activated P-PLB (Thr-17) (upper panel) and T-PLB (lower panel) are shown. The box outlined in black in each panel, indicated by an arrow, corresponds to the cropped part of the blot shown in **Fig. 7A** in the main article.

Supplementary figure 18

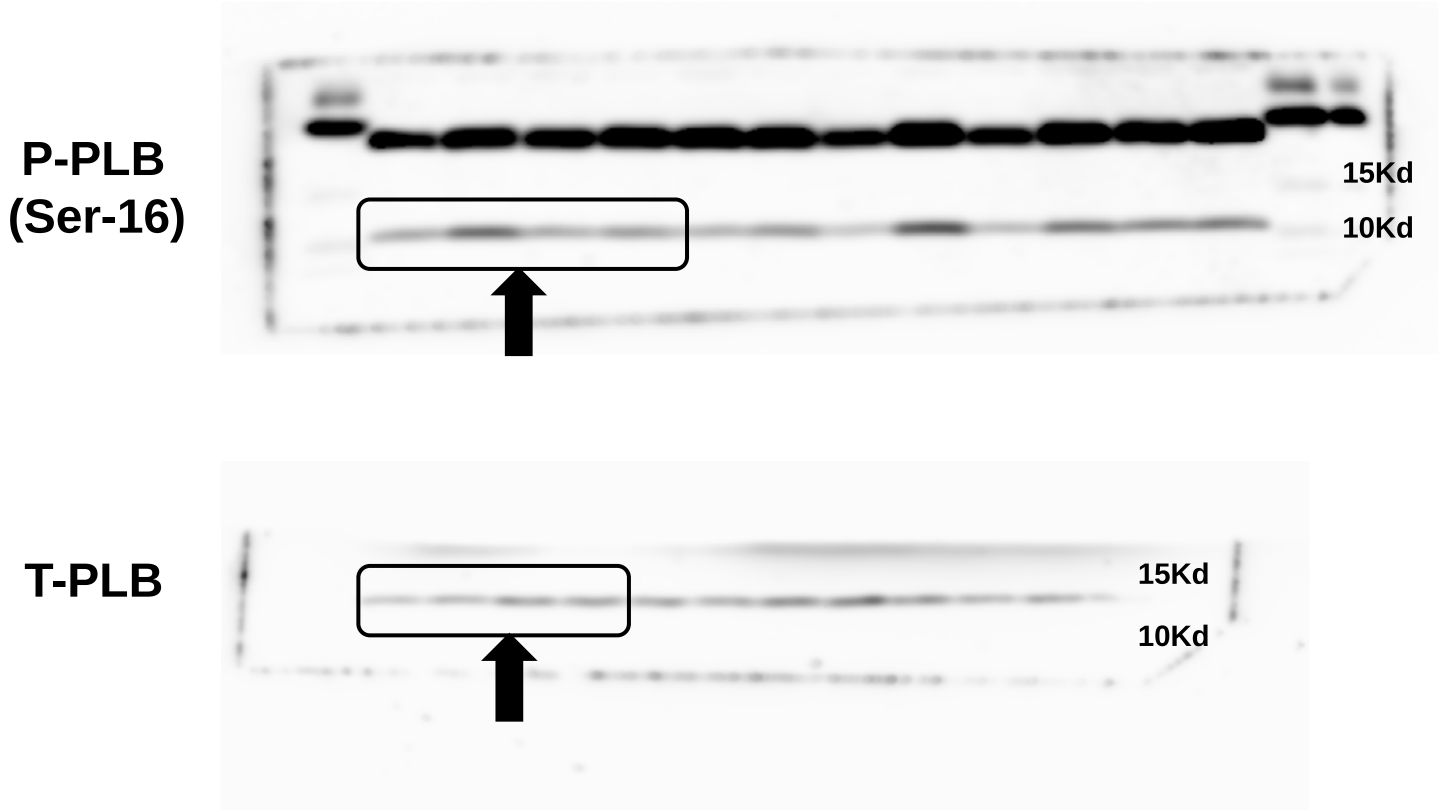

Supplementary Figure 18. Representative full-length western blot.
The amounts of activated P-PLB (Ser-16) (upper panel) and T-PLB (lower panel) are shown. The box outlined in black in each panel, indicated by an arrow, corresponds to the cropped part of the blot shown in **Fig. 7B** in the main article.

Supplementary figure 19

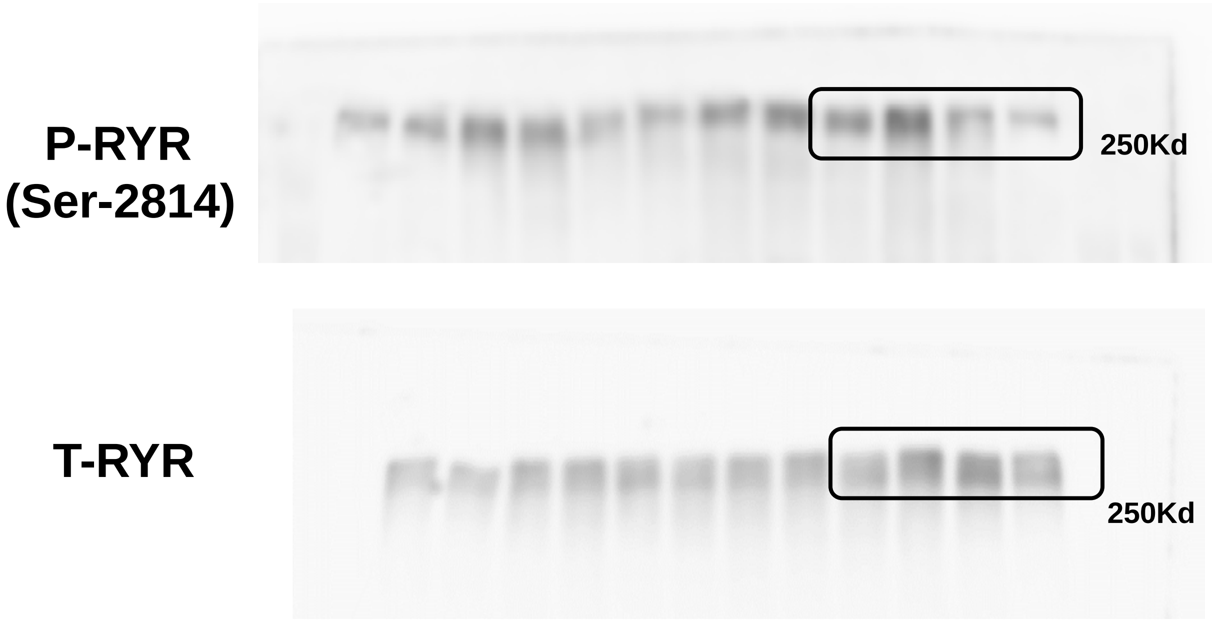

Supplementary Figure 19. Representative full-length western blot (lower panel) are shown. The box outlined in black in each panel, indicated an arrow, corresponds to the cropped part of the blot shown in **Fig. 7C** in the main article.

Supplementary figure 20

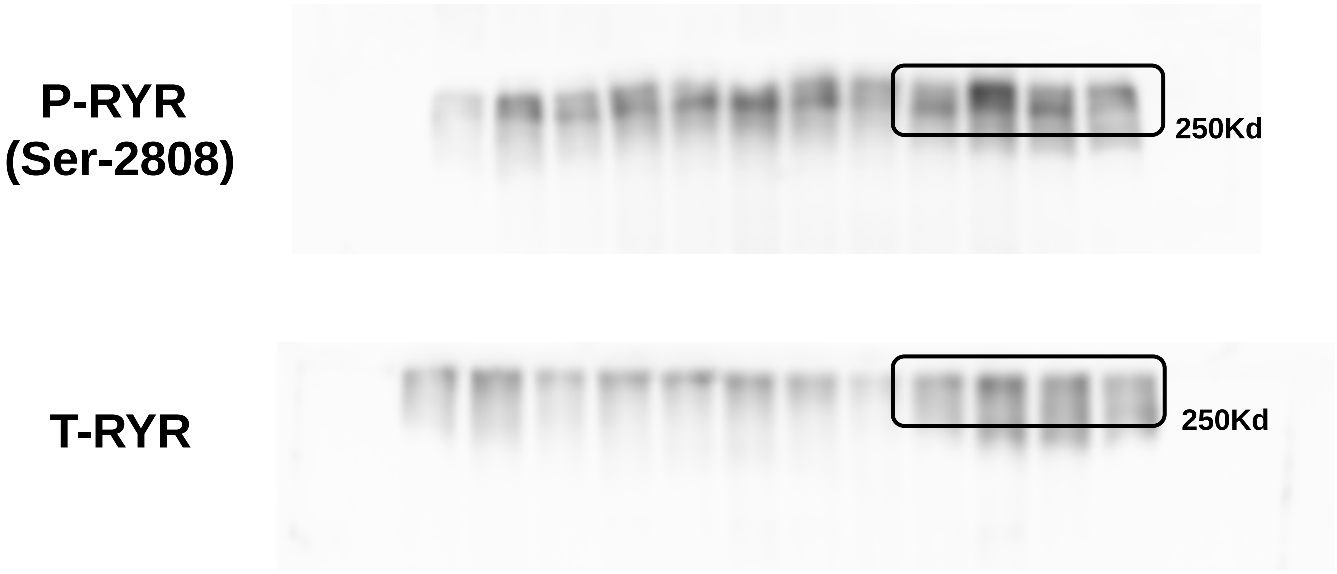

Supplementary Figure 18. Representative full-length western blotting of Fig. 7D. The amounts of activated P-RYR (Ser-2808) (upper panel) and T-RYR (lower panel) are shown. The box outlined in black in each panel, indicated by an arrow, corresponds to the cropped part of the blot shown in **Fig. 7D** in the main article.
